# A treatment planning study comparing Elekta VMAT and fixed field IMRT using the varian treatment planning system eclipse

**DOI:** 10.1186/1748-717X-9-153

**Published:** 2014-07-10

**Authors:** Samuel Peters, Hans Schiefer, Ludwig Plasswilm

**Affiliations:** 1 Department of Radiation Oncology, Kantonsspital St. Gallen, Rorschacherstrasse 95, 9007 St. Gallen, Switzerland

**Keywords:** IMRT, VMAT, Single arc, Double arc, Dose volume histogram, Plan comparison

## Abstract

**Background:**

The newest release of the Eclipse (Varian) treatment planning system (TPS) includes an optimizing engine for Elekta volumetric-modulated arc therapy (VMAT) planning. The purpose of this study was to evaluate this new algorithm and to compare it to intensity-modulated radiation therapy (IMRT) for various disease sites by creating single- and double-arc VMAT plans.

**Methods:**

A total of 162 plans were evaluated in this study, including 38 endometrial, 57 head and neck, 12 brain, 10 breast and 45 prostate cancer cases. The real-life IMRT plans were developed during routine clinical cases using the TPS Eclipse. VMAT plans were generated using a preclinical version of Eclipse with tumor-region-specific optimizing templates without interference of the operator: with one full arc (1A) and with two full arcs (2A), and with partial arcs for breast and prostate with hip implant cases. All plans were evaluated based on target coverage, homogeneity and conformity. The organs at risk (OARs) were analyzed according to plan objectives, such as the mean and maximum doses. If one or more objectives were exceeded, the plan was considered clinically unacceptable, and a second VMAT plan was created by adapting the optimization penalties once.

**Results:**

Compared to IMRT, single- and double-arc VMAT plans showed comparable or better results concerning the target coverage: the maximum dose in the target for 1A is the same as that for IMRT; for 2A, an average reduction of 1.3% over all plans was observed. The conformity showed a statistically significant improvement for both 1A (+3%) and 2A (+6%). The mean total body dose was statistically significant lower for the considered arc techniques (IMRT: 16.0 Gy, VMAT: 15.3 Gy, p < 0.001). However, the sparing of OARs shows individual behavior that depends strongly on the different tumor regions. A clear difference is found in the number of monitor units (MUs) per plan: VMAT shows a reduction of 31%.

**Conclusion:**

These findings demonstrate that based on optimizing templates with minimal interaction of the operator, the Eclipse TPS is able to achieve a plan quality for the Elekta VMAT delivery technique that is comparable to that of fixed-field IMRT. Plans with two arcs show better dose distributions than plans with one arc.

## Introduction

Volumetric-modulated arc therapy (VMAT) is a complex, arc-based treatment technique for intensity-modulated radiation therapy (IMRT). Combining simultaneously varying dose rate, gantry speed and the shape of the multileaf collimator (MLC) aperture, VMAT is the obvious evolution of fixed-field IMRT and intensity-modulated arc therapy (IMAT) delivery [1-9].

The VMAT technique has become clinically and commercially available for both Varian (Varian Medical Systems, Palo Alto, CA, USA) and Elekta (Elekta AB, Stockholm, Sweden) linear accelerators. Different treatment planning systems (TPS) are available for accelerators from both manufacturers, and the VMAT technique produces plan quality and dose distributions that are comparable and often superior to those of fixed-field step-and-shoot or sliding window IMRT for a wide range of disease sites [10-20]. Moreover, the essential advantage of the VMAT technique is the improved efficiency of the treatment in terms of significant reduction of the number of monitor units (MUs) and the shorter delivery time [10,21,22].

VMAT planning for Elekta linear accelerators is supported with the following commercial and clinical routine treatment planning systems: SmartArc as a part of pinnacle^3^ (Philips, Fitchburg WI, USA), Oncentra Masterplan (Nucletron BV (Elekta), Veenendaal, The Netherlands), RayStation (RaySearch Laboratories AB, Stockholm, Sweden) and Monaco (Elekta). To date, it has only been possible to create VMAT plans performed in Eclipse (Varian) using the RapidArc® optimizing algorithm, referred to as the progressive resolution optimizer algorithm (PRO) based on the principle described by Otto [1], for Varian linear accelerators [23]. The newest release of the Eclipse TPS (v. 11.0.39) includes a slightly modified PRO algorithm (129 control points instead of 180 for one full arc) for Elekta VMAT planning [24].

The purpose of this study was to evaluate this algorithm for different disease sites by creating single- and double-arc VMAT plans. Depending on the TPS, achieving acceptable plans is more or less strongly affected by the operator’s experience level. To exclude this qualitative and difficult-to-measure effect, a template based method that allows plans to be generated with almost no user interaction will be introduced. Therefore, the quality of the optimizer can be analyzed. The dynamic arc plans are compared to clinical step-and-shoot IMRT plans on a statistical basis.

## Materials and Methods

### Patient plan selection and planning objectives

From five different tumor regions – head and neck, brain, cervical and endometrial, breast and prostate – 162 fixed-field step-and-shoot IMRT plans that were used for patient treatment between January 2009 and June 2012 on an Elekta Synergy Linac were examined. These patient plans included the following:

– 38 cervical and endometrial cancer cases with a prescribed dose of 45 Gy to the planning target volume (PTV) of the first series planned with 7 fields;

– 57 head and neck (HN) cancer cases with a simultaneously integrated boost (SIB), of which 18 involved adjuvant radiotherapy with a prescribed dose of 64 Gy for PTV1 with one additional dose level (PTV2: 11 × 54 Gy; 7 × 56.1 Gy) planned with 7 fields; the remaining 39 involved definitive radiotherapy with a prescribed dose of 69.3 Gy (PTV1) with one (PTV2: 8 × 54 Gy and 7 × 56.1 Gy) or two additional dose levels (PTV2: 24 × 56.1 Gy and PTV3: 52.8 Gy), all planned with 7 fields;

– 12 brain cancer cases with a prescribed dose of 60 Gy (planned with 5 to 7 fields);

– 10 breast cancer cases with a prescribed dose of 50 Gy, including 6 left and 4 right sided irradiations (planned with 6 to 8 fields);

– 45 prostate cancer cases, of which 18 involved one hip implant and 16 involved two hip implants, both with a prescribed dose of 74 Gy (planned with 5 fields); the remaining 11 cases involved pelvic lymph nodes (LNs) included in the PTV with a prescribed dose of 45 Gy for the first series (planned with 7 fields).

We sought to achieve various planning objectives according to our clinical protocols for PTV and organs at risk (OARs). These values are listed in Table 1. Plans were called “clinically acceptable” if all objectives were met (“in tolerance”). The study was performed based on ethical board approval (Ethics Committee St. Gallen, Switzerland).

**Table 1:** Planning objectives for organs at risk and target volumes

**Organ at risk**	**Maximum dose**	**Dose volume parameter**
Brain	63 Gy	
Brainstem^a^	54 Gy	
Chiasm^b^	53 Gy	
Lens	3 Gy	
Optical nerve^b^	50 Gy	
Lips	45 Gy	
Parotid glands		V_28 Gy_ < 50%
Spine^a^	45 Gy	
Lung		V_20 Gy_ < 20%; D_mean_ < 15 Gy
Lungs, both		V_5 Gy_ < 65%; V_20 Gy_ < 20%; D_mean_ < 13 Gy
Heart		V_30 Gy_ < 20%; D_mean_ < 15 Gy
Small intestine		V_45 Gy_ < 78 cm^3^
Bladder		V_40 Gy_ < 55%; V_60 Gy_ < 25%; V_70 Gy_ < 5%
Rectum		V_40 Gy_ < 60%; V_60 Gy_ < 40%; V_70 Gy_ < 20%
PTV1	110%^c^	V_95%_ > 95%
PTV2 and PTV3 (sub-dose levels)	110%^c^	V_95%_ > 95%; D_50%_ in [98.5% - 101.5%]^c^

Note: PTV = planning target volume; V_95%_ = volume of PTV receiving 95% of prescription; D_50%_ = median dose; V_*n *Gy_ = volume of structure receiving ≥ *n* Gy; ^a^objectives are to be counted including an additional margin of 5 mm, ^b^objectives are to be counted including an additional margin of 3 mm; ^c^in relation to the prescribed dose.

### Treatment planning

#### *Linear accelerator*

All IMRT and VMAT plans were created using the same 6 MV photon beams commissioned for an Elekta Synergy Linac equipped with an MLCi multileaf collimator (40 leaf pairs with 1 cm width, maximum leaf speed of 2.5 cm/s; no interdigitation), maximum gantry speed of 6°/s and variable dose rate up to 500 MU/s (seven fixed dose rate levels were available, each of which was half the dose rate of the next higher level without continuous adjustment).

#### *IMRT*

The clinical step-and-shoot IMRT plans were generated using the Eclipse treatment planning system (version 8.6, Varian Medical Systems). Depending on the tumor region, 5 to 9 coplanar fields were chosen, consistent with our clinical standard (section II.A). The optimizing process was started with standardized optimizing templates (predefined settings of penalty-functions using dose-volume objectives with a certain weight) followed by individual adaptations to achieve the clinical objectives (Table 1). Depending on the tumor region at least 3 (prostate, cervical) to 6 (breast, head and neck, brain) attempts were taken to get a clinically acceptable IMRT plan. The dose calculation was performed using the anisotropic analytic algorithm (AAA) [25] and a grid size of 2.5 mm.

#### *VMAT*

VMAT plans were generated using a preclinical version of the TPS Eclipse (v. 11.0.16). For all corresponding IMRT plans, single-arc (1A) and double-arc plans (2A) were created. Except for the prostate plans with hip implants and breast irradiation, a 358° counter-clockwise rotation for 1A with 129 control points was used; double-arc plans corresponded to two 358° coplanar arcs with the same isocenter in clockwise and counter-clockwise directions. For breast cancer cases, partial arcs of 240° (180° to 300° for left sided targets and 60° to 180° for right sided targets) were used to avoid entrance doses to the contralateral lung. For cases with one hip implant, two resp. four partial arcs were used to avoid direct irradiation of the metal prosthesis; for cases with two hip implants, three and six partial arcs were used. The length of the arcs was adjusted separately for each case (one implant: 60° and 275° on average; two implants: left 55°, middle 76° and right 60°). In all cases, the isocenter was placed in the center of the PTV. The collimator angle was set to 30° for counter-clockwise rotation and to 330° for clockwise rotation. The dose calculation was performed with the AAA algorithm and a grid size of 2.5 mm; for the optimization, the PRO3 algorithm was used.

#### *Optimizing procedure for VMAT*

Based on the available IMRT optimizing templates, new VMAT optimizing templates for each treatment region and prescribed dose were created (in total 10 different templates: cervical, HN 64 Gy/54 Gy, HN 64 Gy/56.1 Gy, HN 69.3 Gy/54 Gy, HN 69.3 Gy/56 Gy, HN 69.3 Gy/56.1 Gy/52.8 Gy, breast, prostate LN, prostate 74 Gy, brain; a description of the templates can be found in the Additional file 1). All these templates were tested on a few patients to evaluate whether they produced reasonable results concerning the plan quality in comparison to IMRT and concerning the irradiation by measuring the treatment time and dose distribution [26]. Using these templates for all corresponding IMRT plans, 1A and 2A plans were created. During the optimizing process, the objectives or weights were not adjusted to exclude the influence of the operator, which allows an objective and independent evaluation of the dose distribution of all tumor regions. After the optimizing process and the final dose calculations, the dose distribution was evaluated according the clinical planning objectives (Table 1) and was compared against the original IMRT plan as a benchmark. If one or more objectives were not met, a second VMAT plan (1Am resp. 2Am) was created starting with the same template. At the beginning of this second optimizing process, the penalties (dose-volume objective and/or weight) corresponding to the clinical objectives out of tolerance were manually adapted based on the experience of IMRT planning. The adapted plans were then evaluated again and no further adapted plans were created, even if any objectives were not met.

### Evaluation

#### *Dose-volume histogram and evaluation parameters*

To quantitatively compare the VMAT plans to the original IMRT plans, dose-volume histograms (DVHs) were used. To visualize the differences, average cumulative DVHs were calculated per examined tumor region for each organ and treatment technique.

Organ-specific individual values such as the mean and maximum dose and partial volume values according to the clinical objectives (Table 1) were derived from the DVHs and average values for each treatment technique and tumor region were calculated, where the average values for 1Am and 2Am include the values of the non-modified plans of the corresponding tumor region. For the PTV beside maximum dose, V_95%_ and values for the homogeneity and conformity were evaluated; the homogeneity index HI was defined as:

(1) $$\mathrm{HI}=\frac{D_{5\%}}{D_{95\%}}$$

where D_5%_ and D_95%_ are the minimum doses received by 5% and 95% of the PTV, respectively; 1 is the smallest and ideal value. The conformity was calculated with the conformation number (van’t Riet model) [27]:

(2) $$\mathrm{CN}=\;\frac{{\mathrm{TV}}_{\mathrm{RI}}\;\cdot \;{\mathrm{TV}}_{\mathrm{RI}}}{\mathrm{TV}\;\cdot \;V_{\mathrm{RI}}}$$

where TV_RI_ is the target volume covered by the reference isodose (95% of prescription), TV is the target volume (PTV) and V_RI_ is the volume of the reference isodose. CN ranges from 0 to 1, where 1 is the ideal value. For sub-dose levels (PTV2, PTV3), the median dose D_50%_ was evaluated. In addition to the above mentioned metrics, the mean body dose, the volume of the body receiving 5 Gy or more (V_5 Gy_) and the number of used MUs per fraction were estimated as a measure for scatter dose.

#### *Statistical analyses*

The results of VMAT and IMRT plans were compared to the two-sided Wilcoxon matched-pair signed-rank test. The threshold for statistical significance was set at *p* < 0.05; a *p*-value less than 0.01 is considered highly significant. The DVH calculations and statistical analyses were performed using MatLab (v 7.7.0.471 [R2008b], The MathWorks Inc., Natick, MA, USA).

## Results

Clinically acceptable VMAT plans showing comparable dose distributions to IMRT plans were achieved for all evaluated tumor regions: clinically acceptable 2A plans were achieved without interference of the operator in 48% of all cases (ranging from 21% for HN 69.3 Gy to 100% for prostate with LN), and acceptable 1A plans were achieved in 31% of all cases (ranging from 16% for endometrial to 73% for prostate with LN). After modifying the optimization penalties of the plans out of tolerance, 62% of the double-arc plans were acceptable, and 41% of the single-arc plans were acceptable. Considering the OARs only, acceptable plans would be achieved in 57% to 75% of cases. For PTV only, double-arc plans would be acceptable in 72% to 73% of cases, and single-arc plans in 46% of cases for 1A and 49% of cases for 1Am. Between the evaluated tumor regions and prescribed doses, differences were identified. The corresponding data are listed in Table 2.

**Table 2:** Ratios of acceptable VMAT plans

	**Number**	**Plan**				**PTV**				**OAR**			
**Site**	**of plans**	**1A**	**1Am**	**2A**	**2Am**	**1A**	**1Am**	**2A**	**2Am**	**1A**	**1Am**	**2A**	**2Am**
*Brain*	12	33%	42%	42%	50%	50%	50%	67%	58%	42%	42%	50%	50%
*Breast*	10	50%	50%	90%	90%	50%	50%	90%	90%	100%	100%	100%	100%
*Endometrial*	38	16%	24%	50%	61%	21%	26%	68%	68%	66%	66%	74%	82%
*HN 64 Gy*	18	33%	44%	44%	56%	61%	67%	94%	100%	44%	61%	50%	61%
*HN 69.3 Gy*	39	21%	28%	21%	36%	41%	44%	44%	49%	38%	46%	38%	59%
*Prostate 1 Impl.*	18	44%	50%	61%	78%	50%	56%	78%	83%	61%	72%	72%	89%
*Prostate 2 Impl.*	16	31%	44%	38%	69%	69%	63%	88%	81%	44%	63%	44%	81%
*Prostate LN*	11	73%	91%	100%	100%	73%	91%	100%	100%	100%	100%	100%	100%
*Total*	162	31%	40%	48%	60%	46%	49%	72%	73%	57%	64%	61%	75%

Before (1A, 2A) and after modification (1Am, 2Am) of the optimization penalties divided by tumor site: on the left, ratios for the total plans are shown; in the middle, ratios for the PTV only; on the right, ratios for the OARs only.

Table 3 summarizes the averaged results in terms of PTV coverage, total body dose and number of MUs of all 162 cases. The mean total body dose was statistically significant lower for all VMAT cases than for IMRT (-0.7 Gy); however, the V_5Gy_ was higher (+0.6%). The number of monitor units was statistically significant lower for VMAT by factors of 1.4 for double-arc and 1.5 for single-arc plans. The PTV coverage for double-arc plans was better than for IMRT (∆D_max_: -1.3%, ∆CN: +0.06, ∆HI: -0.01); V_95%_ revealed no difference. Single-arc plans had clearly lower V_95%_ values (1A: -1.8%, 1Am: -2.0%) and worse homogeneity (+0.01) than IMRT.

**Table 3:** Comparison between IMRT and VMAT for all 162 evaluated patient plans

	**IMRT** ***(n = 162)***	**1A** ***(n = 162)***	**1Am** ***(n = 162)***	**2A** ***(n = 162)***	**2Am** ***(n = 162)***
*PTV*					
D_max_ (%)	108.9 (103.9 - 120.3)	108.8 (104.4 - 119.9)^b^	109.1 (104.8 - 119.7)^c^	107.6 (103.6 - 115.2)^ab^	107.6 (103.6 - 116.0)^ac^
V_95%_ (%)	94.2 (83.3 - 100.0)	92.4 (75.6 - 99.8)^ab^	92.2 (81.8 - 99.6)^ac^	94.2 (75.6 - 99.9)^b^	94.3 (81.0 - 99.9)^c^
HI	1.10 (1.05 - 1.20)	1.11 (1.04 - 1.38)^ab^	1.11 (1.05 - 1.23)^ac^	1.09 (1.04 - 1.35)^ab^	1.09 (1.04 - 1.22)^ac^
CN	0.76 (0.22 - 0.91)	0.79 (0.26 - 0.93)^ab^	0.79 (0.30 - 0.93)^ac^	0.81 (0.29 - 0.94)^ab^	0.82 (0.40 - 0.94)^ac^
*Body*					
D_mean_ (Gy)	16.0 (3.7 - 42.8)	15.2 (3.6 - 39.0)^a^	15.2 (3.6 - 39.9)^a^	15.3 (3.6 - 39.2)^a^	15.3 (3.6 - 40.4)^a^
V_5 Gy_ (%)	31.7 (6.6 - 70.6)	32.1 (6.8 - 69.8)^ab^	32.2 (6.8 - 69.6)^ac^	32.4 (7.0 - 70.1)^ab^	32.4 (7.0 - 70.4)^ac^
*MU*	621.4 (307–1123)	406.9 (261–833)^ab^	424.4 (279–833)^ac^	435.1 (156–939)^ab^	442.2 (156–939)^ac^

Single and double arc plans before (1A, 2A) and after modification (1Am, 2Am) of the optimization penalties; values are expressed as the mean (range).

^a^p < 0.01 for Wilcoxon matched-pair signed rank test vs. IMRT; ^b^p < 0.01 1A vs. 2A; ^c^p < 0.01 1Am vs. 2Am.

In a comparison of single- and double-arc techniques, the PTV coverage was significantly different for plans with two arcs. However, double-arc plans had approximately 6% more MUs than single-arc plans. Differences from 1A to 1Am and 2A to 2Am were not significant, and slight declines were identified for single-arc plans in both maximum dose and V_95%_.

### Cervical and endometrial cases

Of the 38 cervical and endometrial cases for the double-arc technique, 19 were in tolerance for both OAR sparing and PTV coverage; after adapting the optimization penalties, 23 plans met all objectives. For the single-arc technique (1A), six plans were acceptable after adapting (1Am) nine. For most non-acceptable plans, the following values were out of tolerance: D_max_ of the PTV (1A: 10 plans, 2A: 5), V_95%_ (1A: 30 plans, 2A: 10) and V_40 Gy_ of the bladder (1A and 2A: 7 plans).

**Figure 1:**
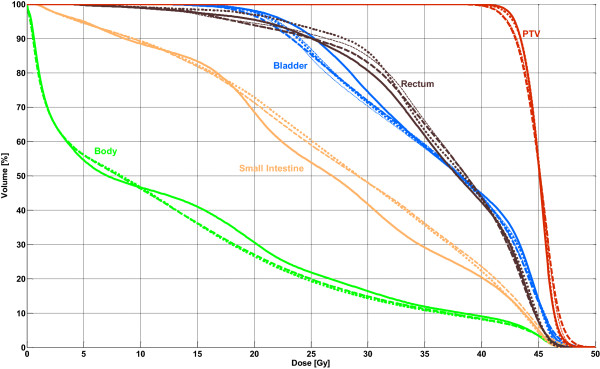
**Mean DVHs of 38 cervical and endometrial cases.** Solid line: IMRT; thin dashed line: 1A; thin dotted line: 2A; fat dashed line: 1Am; fat dotted line: 2Am.

In Table 4, the averaged results in terms of PTV coverage, OAR doses and number of MUs are summarized. The doses in the OARs were similar to IMRT for both single- and double-arc plans; small but significant differences were detected for total body dose (D_mean_: -0.8 Gy; V_5 Gy_: +1.5% for 2A) and D_max_ of the bladder (-0.9 Gy for 2A). Double-arc plans showed a significant reduction of high-dose regions in the small intestine (V_45 Gy_: -4.7 cm^3^), but the mean dose was higher for VMAT (+1.2 Gy). The PTV coverage had highly significant lower V_95%_ values for all arc techniques (IMRT 96.2%, 1A: 92.6%, 2A: 95.2%). The maximum target dose and the conformity were better for double-arc plans than for IMRT; for single-arc plans, the corresponding values were below the mean values of IMRT. The number of MUs for VMAT was statistically significant lower than for IMRT, with reductions ranging from 34% for 2Am to 43% for 1A. Comparing single- and double-arc plans, all parameters (except MUs) exhibited differences in favor of the double-arc technique, and differences in the parameters for PTV coverage, maximum dose in the bladder and rectum were statistically significant. The averaged DVH comparison is plotted in Figure 1.

**Table 4:** Comparison between IMRT and VMAT for 38 cervical and endometrial plans

	**IMRT** ***(n = 38)***	**1A** ***(n = 38)***	**1Am** ***(n = 38)***	**2A** ***(n = 38)***	**2Am** ***(n = 38)***
*PTV*					
D_max_ (%)	109.9 (106.7 - 113.5)	110.2 (107.4 - 117.3)^b+^	111.3 (107.4- 117.3)^ac+^	109.3 (106.2 - 115.2)^ab+^	109.4 (106.2 - 113.0)^c+^
V_95%_ (%)	96.2 (89.5 - 98.7)	92.6 (86.8 - 96.3)^a+b+^	91.9 (83.2 - 96.3)^a+c+^	95.2 (90.6 - 98.4)^a+b+^	95.2 (89.4 - 98.4)^a+c+^
HI	1.09 (1.07 - 1.13)	1.11 (1.09 - 1.16)^a+b+^	1.12 (1.09 - 1.18)^a+c+^	1.09 (1.07 - 1.13)^b+^	1.09 (1.07 - 1.14)^c+^
CN	0.77 (0.65 - 0.89)	0.79 (0.65 - 0.86)^b+^	0.77 (0.62 - 0.85)^c+^	0.83 (0.71 - 0.89)^a+b+^	0.83 (0.68 - 0.89)^a+c+^
*Body*					
D_mean_ (Gy)	13.8 (9.1 - 19.3)	13.1 (8.3 - 17.5)^a+^	13.2 (8.3 - 17.9)^a+c^	13.0 (8.3 - 17.6)^a+b^	13.1 (8.3 - 18.2)^a+c^
V_ 5Gy_ (%)	54.6 (37.4 - 70.6)	55.9 (39.1 - 69.8)^a+b^	56.1 (39.2 - 69.6)^a+^	56.1 (39.5 - 70.1)^a+^	56.1 (39.5 - 70.4)^a+^
*Rectum*					
V_40 Gy_ (%)	42.3 (0.1 - 100.0)	43.9 (0.9 - 100.0)	43.7 (0.1 - 100.0)	44.0 (0.3 - 100.0)	43.3 (0.7 - 100.0)
D_max_ (Gy)	46.9 (41.1 - 49.6)	47.2 (43.8 - 48.8)^b+^	47.2 (42.2 - 49.3)^c+^	46.8 (41.7 - 48.1)^b+^	46.8 (42.0 - 48.7)^c+^
*Bladder*					
V_40 Gy_ (%)	44.7 (7.4 - 100.0)	44.2 (8.6 - 100.0)	43.7 (8.4 - 99.6)	43.8 (6.5 - 100.0)	43.5 (9.4 - 100.0)
D_max_ (Gy)	48.2 (46.3 - 49.6)	47.8 (46.3 - 49.4)^a+b+^	48.1 (46.3 - 50.6)^c+^	47.3 (44.1 - 48.9)^a+b+^	47.4 (44.4 - 49.8)^a+c+^
*Small Intestine*					
V_45 Gy_ (ccm)	29.3 (0.0 - 182.8)	30.2 (0.0 - 161.8)	32.7 (0.1 - 185.5)	24.0 (0.0 - 161.4)^a+^	25.3 (0.0 - 155.7)^a+^
D_mean_ (Gy)	26.8 (10.9 - 36.4)	28.0 (11.3 - 40.6)	28.0 (11.3 - 38.6)	27.9 (11.0 - 39.4)^a^	28.0 (11.0 - 39.5)^a+^
*MU*	701.7 (496–850)	399.7 (324–515)^a+b+^	457.6 (322–641)^a+^	436.6 (356–590)^a+b+^	460.6 (363–621)^a+^

Single and double arc plans before (1A, 2A) and after modification (1Am, 2Am) of the optimization penalties; values are expressed as the mean (range).

^a^p < 0.05 for Wilcoxon matched-pair signed rank test vs. IMRT; ^b^p < 0.05 1A vs. 2A; ^c^p < 0.05 1Am vs. 2Am; ^+^p < 0.01.

### Head and neck cases

Of the 18 adjuvant cases with prescriptions of 64 Gy, for the double-arc technique, 8 plans met the clinical objectives, 10 after modifications. For the single-arc technique, 6 plans met the clinical objectives, 8 plans after modifications. Cases with target doses of 69.3 Gy for definitive radiation therapy had acceptable 2A plans in 8 cases out of 39; after adapting the optimization penalties, the number of acceptable plans was increased to 14; eight single-arc plans were acceptable, 11 after modification. In all non-acceptable plans, either V_95%_ of the PTV1 was too small (1A: 25 plans, 2A: 22) or the objective for V_28 Gy_ in one of the parotid glands was exceeded (1A: 25 plans, 2A: 24); the other values out of tolerance were D_max_ for brainstem (1A: 11 plans, 2A: 5) and spine (1A and 2A: 12 plans).

**Figure 2:**
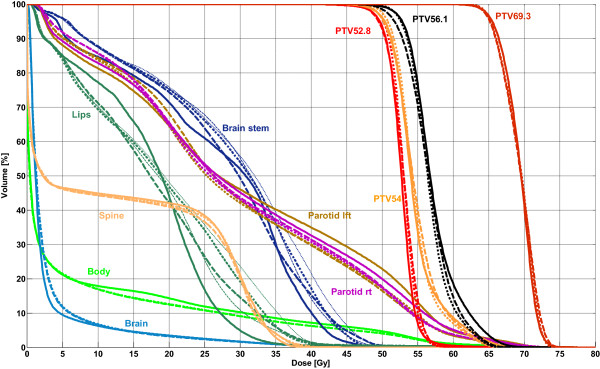
**Mean DVHs of 39 head and neck cancer cases with prescriptions of 69.3 Gy.** Solid line: IMRT; thin dashed line: 1A; thin dotted line: 2A; fat dashed line: 1Am; fat dotted line: 2Am.

Table 5 lists the detailed results for the head and neck cases with prescriptions of 69.3 Gy. Significant differences in IMRT for single- and double-arc plans were noted for total body dose (D_max_: -0.4 Gy; V_5 Gy_: +0.3%). The maximum doses to the lips and the brain exhibited no clear differences between IMRT and VMAT. D_max_ in the brain stem was significantly higher with the VMAT plans (IMRT: 39.3 Gy; 1Am: 42.9 Gy; 2A: 43.1 Gy). However, the maximum spine dose was clearly lower for all VMAT techniques (up to -2.3 Gy). The mean dose and V_28 Gy_ for the parotid glands were slightly better for VMAT after modification of the optimizing process; before modification, they were significantly better for IMRT. The dose coverages in the PTV2 and PTV3 were generally better for VMAT than for IMRT. However, V_95%_ after modification was worse than before modification, particularly for 1Am of 56.1 Gy, where IMRT was significantly better (+5.5%). The median dose of the sub-dose levels was significantly higher than in IMRT (-1.9%); however, after modification, no clear differences were identified. In the 69.3 Gy-target volume, the maximum dose was statistically significant lower (up to -0.8%) for double-arc plans than for IMRT, and the conformity was clearly better for all VMAT techniques (up to +0.08). However, V_95%_ (up to -2.4%) and HI (+0.01) of the VMAT plans were statistically significant below the IMRT values. Comparing the single-arc and double-arc techniques revealed significant differences in favor of double-arc plans concerning target coverage of PTV1 (D_max_: -1.0%, V_95%_: -0.6%, HI: -0.01, CN: +0.02) and the sparing of the spine (D_max_: -0.8 Gy). The averaged DVH comparison of all 39 plans is plotted in Figure 2.

**Table 5:** Comparison between IMRT and VMAT for 39 head and neck plans with a prescription of 69.3 Gy

	**IMRT** ***(n = 38)***	**1A** ***(n = 38)***	**1Am** ***(n = 38)***	**2A** ***(n = 38)***	**2Am** ***(n = 38)***
*PTV 69.3Gy*					
D_max_ (%)	107.6 (103.9 - 110.3)	107.9 (104.4 - 112.7)^b+^	108.0 (105.7-111.8)^ac+^	106.8 (104.4- 109.2)^a+b+^	107.0 (104.4 - 109.9)^c+^
V_95%_ (%)	93.4 (88.1 - 97.8)	91.5 (85.3 - 99.1)^a+^	91.0 (82.9 - 98.1)^a+c^	91.9 (86.1 - 99.1)^a+^	91.6 (85.4 - 98.8)^a+c^
HI	1.10 (1.07 - 1.14)	1.11 (1.06 - 1.15)^a+b+^	1.12 (1.07 - 1.17)^a+c+^	1.10 (1.06 - 1.14)^b+^	1.11 (1.06 - 1.14)^c+^
CN	0.77 (0.22 - 0.91)	0.81 (0.26 - 0.91)^a+b+^	0.83 (0.30 - 0.92)^a+c+^	0.84 (0.29 - 0.93)^a+b+^	0.85 (0.40 - 0.93)^a+c+^
*PTV 56.1Gy*					
D_max_ (%)	127.8 (124.0 - 135.1)	122.7 (115.6 - 130.4)^a+^	123.2 (116.7-129.0)^a+c+^	122.3 (117.0 - 126.4)^a+^	122.1 (118.2-127.2)^a+c+^
V_95%_ (%)	93.1 (81.7 - 97.9)	93.3 (85.5 - 97.3)^b+^	87.6 (80.3 - 94.9)^a+c+^	96.1 (92.2 - 99.1)^a+b+^	92.0 (84.1 - 97.2)^c+^
HI	1.21 (1.12 - 1.31)	1.19 (1.14 - 1.24)^ab+^	1.21 (1.14 - 1.28)^c+^	1.16 (1.10 - 1.22)^a+b+^	1.18 (1.10 - 1.25)^a+c+^
CN	0.67 (0.46 - 0.82)	0.69 (0.42 - 0.83)^b+^	0.69 (0.49 - 0.81)^c+^	0.71 (0.44 - 0.85)^a+b+^	0.73 (0.50 - 0.85)^a+c+^
D_50%_ (%)	101.2 (99.0 - 104.3)	102.6 (101.1-105.9)^a+b+^	100.8 (99.5 - 102.1)	102.2 (101.0 -104.6)^a+b+^	100.6 (98.9 - 101.9)^a^
*PTV 54Gy*					
D_max_ (%)	129.9 (106.5 - 136.3)	126.8 (114.5 - 134.6)^ab+^	126.8 (112.5 - 133.0)^a^	124.5 (110.7 -129.8)^a+b+^	125.6 (109.1 - 132.9)^a+^
V_95%_ (%)	90.1 (85.0 - 95.3)	94.0 (90.8 - 97.5)^a+b+^	89.0 (79.2 - 92.9)	96.1 (92.5 - 98.7)^a+b+^	92.5 (87.3 - 97.5)
HI	1.22 (1.09 - 1.28)	1.20 (1.12 - 1.25)^b+^	1.21 (1.12 - 1.31)^c+^	1.17 (1.09 - 1.24)^a+b+^	1.19 (1.09 - 1.27)^a+c+^
CN	0.70 (0.57 - 0.79)	0.73 (0.63 - 0.87)^a+b+^	0.74 (0.65 - 0.81)^a+^	0.76 (0.66 - 0.88)^a+b+^	0.76 (0.65 - 0.87)^a+^
D_50%_ (%)	100.1 (98.4 - 101.5)	102.4 (101.3-103.4)^a+b+^	100.6 (98.9 - 102.2)	101.9 (101.1- 103.2)^a+b+^	100.5 (99.8 - 101.8)
*PTV 52.8Gy*					
D_max_ (%)	114.0 (103.9 - 134.7)	114.9 (109.6 - 128.2)^b+^	112.9 (108.0 - 126.5)	113.1 (109.1 - 127.3)^b+^	112.0 (106.2 - 128.3)
*V*_95%_ (%)	92.7 (73.0 - 99.5)	96.5 (91.7 - 99.3)^ab+^	91.9 (83.8 - 97.9)^c+^	97.9 (95.5 - 99.8)^a+b+^	95.1 (87.1 - 99.2)^c+^
HI	1.11 (1.07 - 1.21)	1.13 (1.09 - 1.18)^b+^	1.13 (1.09 - 1.18)^c+^	1.10 (1.07 - 1.14)^b+^	1.11 (1.07 - 1.15)^c+^
CN	0.64 (0.47 - 0.75)	0.71 (0.63 - 0.84)^a+^	0.72 (0.64 - 0.87)^a+c^	0.72 (0.64 - 0.84)^a+^	0.74 (0.63 - 0.88)^a+c^
D_50%_ (%)	99.9 (98.0 - 102.7)	102.7 (101.5-104.5)^a+b+^	100.5 (99.5 - 101.5)	102.2 (101.0 -103.6)^a+b+^	100.5 (99.1 - 101.8)
*Body*					
D_mean_ (Gy)	7.1 (3.9 - 18.2)	6.7 (3.7 - 17.0)^a+^	6.6 (3.6 - 16.8)^a+c+^	6.7 (3.7 - 17.0)^a+^	6.6 (3.6 - 16.6)^a+c+^
V_5Gy_ (%)	21.2 (10.6 - 56.6)	21.4 (10.7 - 58.2)^a+b+^	21.4 (10.7 - 57.5)^a+c+^	21.6 (10.7 - 58.5)^a+b+^	21.5 (10.8 - 58.7)^a+c+^
*Brain*					
D_max_ (Gy)	38.8 (3.7 - 62.9)	41.5 (5.2 - 60.4)^ab^	40.1 (5.1 - 58.6)	40.1 (5.2 - 63.3)^b^	39.9 (5.0 - 60.7)
*Brain stem*					
D_max_ (Gy)	39.3 (20.6 - 51.0)	44.6 (22.2 - 51.5)^a+^	42.9 (18.9 - 52.5)^a^	44.3 (15.0 - 51.6)^a+^	43.1 (17.0 - 51.0)^a^
*Lips*					
D_max_ (Gy)	28.6 (2.1 - 71.4)	29.1 (2.4 - 70.4)	28.3 (2.2 - 64.7)	30.4 (2.2 - 70.3)	29.8 (2.2 - 69.3)
*Parotid gland*					
D_mean_ (Gy)	30.2 (7.8 - 62.7)	30.7 (9.7 - 55.7)^a+b+^	29.5 (9.7 - 55.8)	30.3 (9.6 - 55.7)^ab+^	29.2 (9.8 - 56.0)
V_28Gy_ (%)	48.1 (8.7 - 100.0)	49.6 (11.0 - 92.9)^a+b+^	46.6 (12.1 - 93.0)	48.2 (10.3 - 93.2)^ab+^	45.6 (10.4 - 93.3)
*Spine*					
D_max_ (Gy)	37.4 (33.1 - 44.3)	36.3 (33.2 - 44.6)^ab+^	36.1 (32.7 - 44.0)^ac+^	35.1 (32.5 - 41.0)^a+b+^	35.3 (32.6 - 42.8)^a+c+^
*MU*	649.2 (458–802)	370.5 (313–524)^a+b+^	372.9 (326–482)^a+c+^	393.1 (322–558)^a+b+^	393.6 (320–538)^a+c+^

Single and double arc plans before (1A, 2A) and after modification (1Am, 2Am) of the optimization penalties; values are expressed as the mean (range).

^a^p < 0.05 for Wilcoxon matched-pair signed rank test vs. IMRT; ^b^p < 0.05 1A vs. 2A; ^c^p < 0.05 1Am vs. 2Am; ^+^p < 0.01.

The averaged results for head and neck cases with target doses of 64 Gy were as follows. The mean total body dose was similar for IMRT and VMAT; however, the V_5Gy_ value was lower for IMRT (-0.6%) than for double-arc plans. The maximum doses in the brain and the spine and the mean dose of the parotid glands showed no difference between IMRT and VMAT. The maximum dose in the lips was statistically significant lower for VMAT (up to -6.0 Gy); however, the dose sparing in the brain stem was better for IMRT (IMRT: 35.3 Gy; 1A: 42.7 Gy; 2A: 40.5 Gy). The dose coverage in the target volumes was better for all VMAT techniques. Significant differences were noted for the maximum doses for both dose levels (PTV1 > 1.5%, PTV2 > 3.9%), for V_95%_ (PTV1 for 2Arcs: 4.2%, PTV2: 4.4% (1A) and 6% (2A)) and for CN of PTV1 (1A: 0.08, 2A: 0.12). Comparing the single- and double arc techniques reveals significant differences in favor of double-arc plans concerning target coverage of PTV1 (D_max_: -0.9%, V_95%_: +2.3%, HI: -0.02, CN: +0.04), sparing of the brain (D_max_: -1.8 Gy) and the brain stem (D_max_: -1.4 Gy). Detailed values and the averaged DVH comparison are shown in the Additional file 2.

### Breast cases

An acceptable plan was achieved for the double-arc technique in 9 out of 10 breast cancer cases; an acceptable plan was achieved in 5 cases for the single-arc technique. In all non-acceptable cases (before and after adapting the optimizing process), either the maximum target dose was too high (1A: 2 plans, 1Am: 3) or the V_95%_ was too small (1A: 5 plans; 1Am: 3 plans; 2A and 2Am: 1 plan). The OARs met the clinical objectives in all cases for all techniques.

For both single- and double-arc plans, the OARs and total body dose showed slight but non-significant improvements compared to IMRT; however, the maximum spine dose (1A: +1.9 Gy, 2A: +1.1 Gy, p > 0.05) and sparing the contralateral lung with two arcs (V_5 Gy_: +2.1%, D_mean_: +0.2 Gy, p > 0.05) were better for IMRT. Significant differences in IMRT in favor of VMAT were only noted in the ipsilateral lung for double-arc plans (V_5 Gy_: -7.5%, V_20 Gy_: -2.5%, D_mean_: -2 Gy). The mean dose for the heart seems to show a statistically significant improvement for single arc plans (IMRT: 15.9 Gy, 1A 12.6 Gy, 1Am 12.8 Gy, p: 0.039). But the differences depend on the side of the irradiated breast: for left-sided breast irradiation, VMAT showed a slight improvement (IMRT: 18.5 Gy, 1A: 12.7 Gy, 1Am: 12.8 Gy), whereas for right-sided breast irradiation, no differences were noted (IMRT: 12.6 Gy, 2A: 13.0 Gy, 2Am: 12.9 Gy). Due to the low number of cases (6 left, 4 right), no statistical significance for the differences can be given. VMAT plans with two arcs provided significantly better target coverage than IMRT (D_max_: -7.5%; V_95%_: +2.7%; HI: -0.03; CN: +0.06), while single-arc plans showed no clear difference compared to IMRT (except for D_max_: -3.3% for 1A). A comparison of single- and double-arc techniques revealed significant differences in favor of double-arc plans concerning target coverage (D_max_: -4.7%, V_95%_: -4.0%, HI: -0.04), whereas there were no differences in terms of OAR sparing. Detailed values and the averaged DVH comparison are shown in the Additional file 3.

### Prostate cases

A total of 45 prostate cases were analyzed, of which 11 involved pelvic lymph nodes, 16 involved one hip implant and 18 involved two hip implants. All 2A plans for the LN cases met the clinical objectives; 1A plans were acceptable in 8 cases, 10 after modification. Double-arc plans with one implant were acceptable in 11 of the 18 total cases and in 14 cases after adapting the optimization penalties. For the single-arc technique, 8 plans met the objectives, 9 after modification. Cases with two implants had acceptable 2A plans in 6 of 16 cases. After modifying the optimization penalties, the number of acceptable plans increased to 11; 5 single-arc plans were acceptable, 7 after modification.

For prostate cases with LNs, the sparing of the OARs for both single- and double-arc plans were not different compared to IMRT; however, in VMAT plans, the maximum dose in the rectum was smaller (1A: -1.0 Gy, 1Am: -0.8 Gy, 2A: -0.8 Gy). The mean total body dose for VMAT was statistically significant lower than for IMRT (-0.5 Gy), but in VMAT plans, the volume receiving 5 Gy was larger (up to +2.8%). The target coverage was significantly better for double-arc VMAT than for IMRT (D_max_: -3.5%; V_95%_: -1.9%, HI: -0.02, CN: +0.10). Single-arc plans had smaller improvements in comparison to IMRT, with significant differences only for D_max_ (-2.0%) and conformity (+0.06). A comparison of single- and double-arc plans revealed highly significant differences in favor of 2A in target coverage and the maximum bladder dose. The Additional file 4 shows detailed results and the averaged DVH comparison.

Prostate cases with one hip implant presented varying results depending on the treatment technique. The bladder dose for VMAT plans was improved in comparison to IMRT, where V_60 Gy_ was statistically significant lower (-2.8%); the rectum dose was worse, as the maximum dose (up to +1.3 Gy) and V_40 Gy_ (up to +6.6%) were significantly different. The mean dose in the femoral head was statistically significant lower for IMRT in comparison to both VMAT techniques (+3.6 Gy), but the sparing of the implant was clearly better for VMAT (D_max_: -6.7 Gy, D_mean_: -0.7 Gy). Single-arc plans had significantly worse target coverage than IMRT (D_max_ +1.8G%, V_95%_: -2.0%, HI: +0.01); double-arc plans were slightly worse than IMRT but were not significantly different. However, conformity was significantly better for single- and double-arc plans (CN: +0.07). Here, we identified the only cases in which VMAT had more MUs than IMRT; however, the differences (16 MUs on average) were not significant. The differences between single- and double-arc plans were small: significant differences in favor of 2A were identified for target coverage (D_max_ -0.9 Gy, V_95%_: -1.6%, HI: -0.01; CN: +0.01). The total body and rectum doses were better in favor of single-arc plans. Detailed values and the averaged DVH comparison are shown in the Additional file 5.

**Figure 3:**
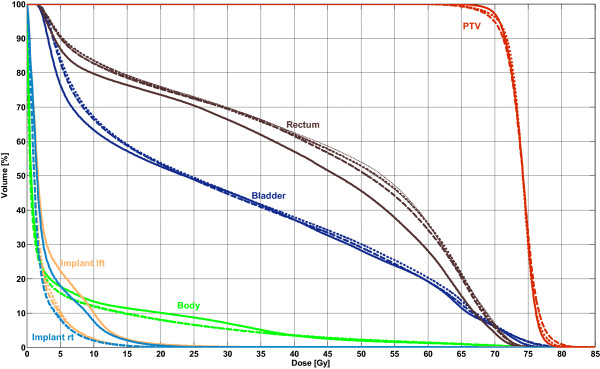
**Mean DVHs of 18 prostate cancer cases with two hip implants.** Solid line: IMRT; thin dashed line: 1A; thin dotted line: 2A; fat dashed line: 1Am; fat dotted line: 2Am.

Table 6 lists the averaged values for OAR doses, target coverage and the number of MUs for prostate cases with two hip implants. The sparing of the OARs for both single- and double-arc plans was slightly worse compared to IMRT; the V_40 Gy_ (up to +5.5%) and V_60 Gy_ (up to +8.1%) for the rectum and the D_max_ of the bladder (up to +1.7 Gy) were significantly different. However, the sparing of the implants and the total body dose were significantly reduced in comparison to the IMRT plans (implant: D_max_: -12.3 Gy, D_mean_: -1.3 Gy; body: D_mean_: -0.6 Gy; V_5 Gy_: -1.9%). The target coverage for VMAT was worse than for IMRT, where single-arc plans were significantly different in D_max_ (up to +2.5%). The conformity was clearly better for the IMRT plans (CN: +0.07). The number of MUs was statistically significant lower for single-arc plans than for IMRT by a factor of 1.2; the double-arc plans had slightly less MUs than IMRT (-7%). The differences between single- and double-arc plans were small; significant differences in favor of 2A were noted for the maximum dose in the PTV (-1.2%) and for the homogeneity (-0.01). The averaged DVH comparison between the considered techniques is plotted in Figure 3.

**Table 6:** Comparison between IMRT and VMAT for 18 prostate cases with two hip implants

	**IMRT** ***(n = 18)***	**1A** ***(n = 18)***	**1Am** ***(n = 18)***	**2A** ***(n = 18)***	**2Am** ***(n = 18)***
*PTV*					
D_max_ (%)	106.6 (104.2 - 112.5)	108.8 (104.9 - 115.4)^ab+^	109.1 (105.5 - 119.7)^ac^	107.6 (104.9 - 113.4)^b+^	108.1 (104.9 - 116.0)^c^
V_95%_ (%)	96.3 (89.2 - 100.0)	94.6 (85.4 - 99.6)	93.9 (82.7 - 99.6)^a^	96.2 (86.5 - 99.8)	95.6 (84.3 - 99.8)
HI	1.09 (1.05 - 1.15)	1.10 (1.05 - 1.20)^b+^	1.11 (1.05 - 1.23)^c+^	1.09 (1.04 - 1.19)^b+^	1.09 (1.04 - 1.22)^c+^
CN	0.78 (0.63 - 0.86)	0.71 (0.46 - 0.82)^a^	0.72 (0.44 - 0.82)^a^	0.71 (0.46 - 0.81)^a^	0.71 (0.45 - 0.81)^a^
*Body*					
D_mean_ (Gy)	5.3 (3.9 - 9.0)	4.7 (3.4 - 7.2)^a+^	4.7 (3.4 - 7.0)^a+^	4.7 (3.4 - 7.2)^a+^	4.7 (3.4 - 7.1)^a+^
V_5 Gy_ (%)	17.7 (11.4 - 29.1)	15.8 (10.3 - 23.4)^a^	15.8 (10.3 - 21.9)^a^	15.8 (10.3 - 23.2)^a^	15.7 (10.2 - 22.4)^a^
*Rectum*					
V_40 Gy_ (%)	57.0 (38.9 - 70.1)	62.2 (55.9 - 76.5)^a+^	61.5 (55.9 - 72.7)^a^	62.5 (57.4 - 78.9)^a+^	61.9 (57.4 - 75.8)^a^
V_60 Gy_ (%)	28.1 (18.8 - 42.3)	35.8 (14.7 - 53.7)^a+^	34.2 (13.9 - 51.5)^a+^	36.2 (21.2 - 56.4)^a+^	36.0 (21.2 - 54.8)^a+^
V_70 Gy_ (%)	4.2 (0.0 - 12.7)	6.6 (0.0 - 24.4)	5.7 (0.0 - 19.8)^c^	7.0 (0.0 - 25.5)^a+^	6.3 (0.0 - 21.3)^a+c^
D_max_ (Gy)	72.5 (66.7 - 77.7)	73.5 (68.8 - 78.3)	73.4 (68.0 - 78.4)	73.3 (68.1 - 77.5)	73.4 (68.2 - 77.5)
*Bladder*					
V_40 Gy_ (%)	37.2 (20.5 - 76.5)	37.9 (23.0 - 68.1)	37.1 (23.0 - 63.9)	37.5 (24.2 - 63.1)	37.9 (24.2 - 69.7)
V_60 Gy_ (%)	19.1 (9.3 - 38.2)	20.0 (10.3 - 38.9)	19.2 (10.9 - 40.7)	20.4 (11.7 - 40.4)	20.3 (10.3 - 40.3)
V_70 Gy_ (%)	6.1 (0.0 - 21.3)	6.5 (0.0 - 25.5)	6.0 (0.0 - 27.5)	7.0 (0.0 - 27.8)	6.5 (0.0 - 28.2)
D_max_ (Gy)	73.9 (67.4 - 79.9)	75.6 (69.0 - 83.7)^a+^	75.4 (69.9 - 83.8)^a^	75.0 (69.5 - 81.9)^a^	74.9 (68.1 - 81.5)^a+^
*Implants*					
D_max_ (Gy)	25.6 (4.3 - 74.4)	13.2 (3.5 - 66.7)^a+^	13.3 (3.6 - 67.4)^a+^	13.3 (3.3 - 67.7)^a+^	13.4 (3.5 - 66.7)^a+^
D_mean_ (Gy)	3.2 (0.9 - 8.1)	1.9 (0.7 - 6.0)^a+^	1.9 (0.7 - 6.0)^a+^	1.9 (0.7 - 6.6)^a+^	1.9 (0.7 - 6.6)^a+^
*MU*	585.3 (435–761)	488.1 (436–595)^ab^	491.4 (424–595)^ac+^	536.0 (410–701)^b^	556.1 (415–744)^c+^

Single and double arc plans before (1A, 2A) and after modification (1Am, 2Am) of the optimization penalties; values are expressed as the mean (range).

^a^p < 0.05 for Wilcoxon matched-pair signed rank test vs. IMRT; ^b^p < 0.05 1A vs. 2A; ^c^p < 0.05 1Am vs. 2Am; ^+^p < 0.01.

### Brain cases

Of the 12 brain cancer cases, five 2A plans were acceptable for both OAR sparing and target coverage; after adapting the optimization penalties, 6 plans met all objectives. For single-arc technique, 4 plans were accepted, 5 after adaptation. In the non-acceptable plans, in general, either V_95%_ was too small (1A: 6 plans; 1Am: 5; 2A and 2Am: 4 plans) or the lens dose exceeded the tolerance (1A: 6 plans; 1Am: 5 plans; 2A: 6 plans, 2Am: 5 plans).

**Figure 4:**
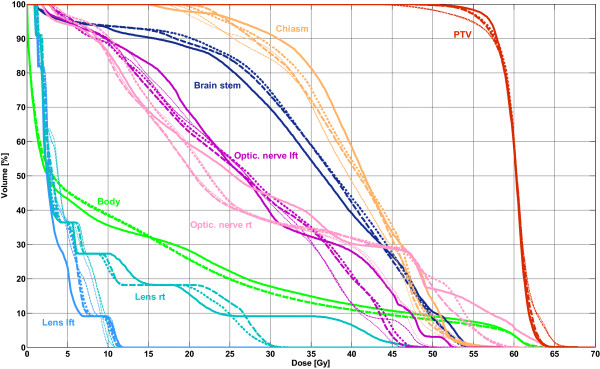
**Mean DVHs of 12 brain cancer cases.** Solid line: IMRT; thin dashed line: 1A; thin dotted line: 2A; fat dashed line: 1Am; fat dotted line: 2Am.

Table 7 summarizes the average values for OAR doses, target coverage and number of MU. The D_max_ of the brain stem was higher for VMAT than for IMRT (up to +3.5 Gy); similar findings were obtained for the chiasm (up to +1.8 Gy). However, the maximum doses of the lenses and the optical nerves were lower for VMAT (lenses: up to -1.8 Gy; opt. nerve: up to -3.4 Gy); after modification, the D_max_ of the right lens changed from 5.8 Gy to 9.0 Gy (IMRT: 10.5 Gy). For the right optical nerve, increases of 2.0 Gy to 40.8 Gy for 2Am and 0.8 Gy to 39.8 Gy for 1Am (IMRT: 42.3 Gy) were observed. The maximum dose in the PTV and the conformity were slightly better for VMAT; however, the homogeneity and V_95%_ were better for IMRT. Significant differences were only observed for conformity in both modified single- and double-arc plans (CN: +0.06). Differences between the two VMAT techniques were small and could be observed mainly in the PTV after modification (D_max_: -0.9%, HI: -0.01, CN: +0.01) and in the total body dose (D_mean_ -0.1 Gy, V_5 Gy_: 0.2%). The DVH comparison of these techniques is plotted in Figure 4.

**Table 7:** Comparisons between IMRT and VMAT for 12 brain cases

	**IMRT** ***(n = 12)***	**1A** ***(n = 12)***	**1Am** ***(n = 12)***	**2A** ***(n = 12)***	**2Am** ***(n = 12)***
*PTV*					
D_max_ (%)	108.1 (104.3 - 113.0)	107.8 (104.8 - 115.5)	107.2 (104.8 - 110.4)^c+^	107.0 (103.6 - 112.8)	106.3 (103.6 - 109.0)^a+c+^
V_95%_ (%)	95.3 (90.6 - 99.3)	91.0 (75.6 - 99.2)	92.2 (83.5 - 99.2)	91.1 (75.6 - 99.8)	93.0 (81.0 - 99.8)
HI	1.09 (1.05 - 1.13)	1.14 (1.05 - 1.38)	1.11 (1.05 - 1.18)^c^	1.13 (1.04 - 1.35)	1.10 (1.04 - 1.19)^c^
CN	0.78 (0.72 - 0.86)	0.80 (0.57 - 0.93)	0.83 (0.67 - 0.93)^ac^	0.81 (0.56 - 0.94)	0.84 (0.65 - 0.94)^ac^
*Body*					
D_mean_ (Gy)	13.5 (3.8 - 21.9)	13.2 (4.4 - 21.0)	13.1 (4.4 - 20.7)^c^	13.2 (4.3 - 20.9)	13.2 (4.4 - 20.9)^c^
V_5Gy_ (%)	43.2 (17.7 - 62.1)	45.3 (20.8 - 63.7)^a+^	45.3 (21.1 - 62.0)^ac^	45.4 (21.1 - 62.9)^a+^	45.5 (21.0 - 62.4)^a+c^
*Brain stem*					
D_max_ (Gy)	50.0 (43.5 - 56.6)	53.1 (51.4 - 56.7)^a^	53.6 (51.3 - 56.7)^ac^	52.8 (51.0 - 55.1)	52.9 (51.0 - 55.1)^c^
Lenses					
D_max_ (Gy)	7.3 (1.0 - 49.3)	5.5 (1.5 - 11.8)	7.0 (1.5 - 31.2)^a^	5.7 (1.5 - 11.3)	6.9 (1.5 - 32.1)
*Lens lft*					
D_max_ (Gy)	4.2 (1.2 - 11.9)	5.4 (1.5 - 11.8)^a+^	4.9 (1.5 - 11.7)^a+^	5.5 (1.5 - 10.6)^a^	4.8 (1.5 - 11.6)^a+^
Lens rt					
D_max_ (Gy)	10.5 (1.0 - 49.3)	5.7 (1.5 - 11.1)	9.1 (1.5 - 31.2)	5.8 (1.5 - 11.3)	9.0 (1.5 - 32.1)
*Chiasm*					
D_max_ (Gy)	50.1 (43.2 - 56.7)	50.9 (32.7 - 60.7)^b^	51.9 (36.5 - 60.7)	50.2 (29.8 - 60.3)^b^	51.4 (33.4 - 60.3)
*Optical nerves*					
*D*_max_ (Gy)	43.2 (25.8 - 64.4)	40.2 (18.5 - 54.1)	41.0 (19.0 - 59.7)	39.8 (19.0 - 52.8)^a^	41.5 (19.7 - 60.0)
*Optical nerve lft*					
D_max_ (Gy)	42.3 (27.3 - 52.6)	41.4 (29.3 - 50.4)	42.1 (31.4 - 47.8)	40.9 (29.2 - 46.6)	42.3 (30.0 - 49.6)
*Optical nerve rt*					
D_max_ (Gy)	44.1 (25.8 - 64.4)	39.0 (18.5 - 54.1)	39.8 (19.0 - 59.7)	38.8 (19.0 - 52.8)^a^	40.8 (19.7 - 60.0)
*MU*	454.0 (330–775)	303.0 (261–342)^a+^	302.6 (279–342)^a^	293.2 (156–331)^a+^	293.7 (156–331)^a+^

Single and double arc plans before (1A, 2A) and after modification (1Am, 2Am) of the optimization penalties; values are expressed as the mean (range).

^a^p < 0.05 for Wilcoxon matched-pair signed rank test vs. IMRT; ^b^p < 0.05 1A vs. 2A; ^c^p < 0.05 1Am vs. 2Am; ^+^p < 0.01.

## Discussion

The question of whether dose distribution and quality are different in VMAT plans compared to IMRT plans has been evaluated in a large number of studies [10-20]. Even if the results of these studies show some differences that may be caused by different optimizing engines, the number of arcs per plan, the linac type or tumor sites, the conclusions are consistent: the plan quality does not clearly differ between IMRT and VMAT. The main difference between the two techniques, and therefore an advantage for VMAT, lies in the reduction in delivery time. This study, in which Elekta VMAT plans generated with Eclipse TPS from Varian are compared with IMRT, supports this statement (IMRT: in average 99 seconds/field; VMAT: in average 82 seconds/arc [26]). However, in contrast to other studies, the primary goal in this study was not to generate “perfect” plans. With the introduced procedure of the optimizing process, the influence of the operator was eliminated or at least minimized. Therefore, the proper quality of the optimizing engine is shown.

In summary, as shown in Table 2, it is possible to create clinically acceptable and IMRT-comparable plans based on templates using this VMAT optimizer for all tumor regions. With the use of templates, acceptable plans are easy to generate and minimal operator experience or interaction is required. The calculation times – often a disadvantage of VMAT planning [9,10,13] – do not show large differences: the time to optimize and calculate single-arc plans is approximately 10 to 20% shorter than for IMRT; for double arc plans, the time is 10 to 20% longer.

Regarding the individual tumor regions and dose prescriptions, clear differences in the plan quality in comparison to IMRT and between single- and double-arc plans were identified. However, the values listed in Tables 4, 5, 6 and 7 and in the Additional files 2, 3, 4 and 5 should be carefully interpreted based on the clinical context. Even when statistical significance was noted, the differences could be very small (e.g., V_20 Gy_ for total lung in breast cases: 10.3% for IMRT and 9.5% for 2Am-VMAT, p: 0.027), or the differences were large but the values were still within the tolerance range (e.g., D_max_ of brain stem for 69.3 Gy-HN cases: 35.3 Gy for IMRT and 39.4 Gy for 2Am-VMAT, p: 0.014).

In the HN cases with SIB (64 Gy and 69.3 Gy), the change in the percentage of plans with acceptable target coverage before and after modification of the optimization penalties was small (5%). However, the OAR sparing was substantially improved (+21%). This finding may indicate that the templates used were not ideal, as confirmed by the analyses of the out-of-tolerance parameters (mainly V_28 Gy_ of the parotid glands). However, the target coverage of the 69.3 Gy plans was generally worse than in other tumor regions and dose prescriptions. This finding indicates that in complex cases with up to three different dose levels (PTV1, PTV2, PTV3), the use of a generally valid template cannot be expected to produce an ideal dose distribution without further individual adaptations. Therefore, more effort is needed, particularly to achieve an acceptable V_95%_ value within the PTV1.

Plans with brain tumors showed similar behavior: the acceptance rate was low (<50%). After modifying the optimization penalties, no improvements could be identified for either the target volume or the OAR. In these cases, small and fine structured contours such as lenses and optical nerves often lie near to the target. During irradiation with the VMAT technique, due to the rotation around the body, these OARs were effectively always in the beam path. The MLCi of a leaf width of 1 cm without the possibility of interdigitation used in this study limits the modulation to spare these small structures. MLCs with smaller widths may improve the plan quality for such cases [28-30]. IMRT also improves plan quality; the fixed gantry angles can be placed systematically to avoid irradiation through certain regions with such small structures.

**Figure 5:**
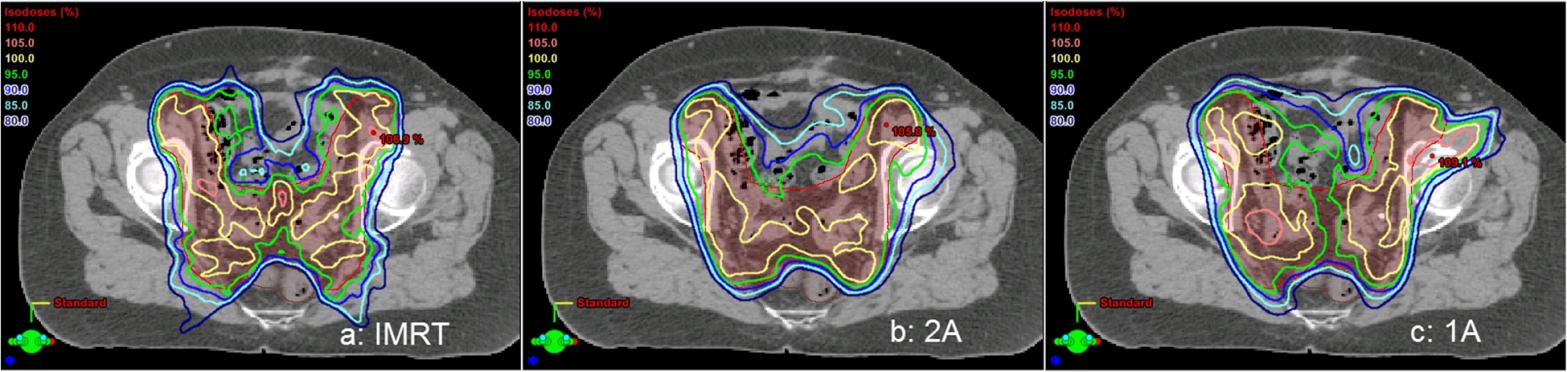
**Example of an endometrial plan showing the differences in target coverage and conformity.** PTV in red and rectum in brown: **(a)** IMRT: CN 0.73, V_95%_ 96.1%, D_max_ 109.7%; **(b)** 2A: CN 0.83, V_95%_ 95.1%, D_max_ 110.6%; **(c)** 1A: CN 0.78, V_95%_ 92.2%, D_max_ 112.4%.

The endometrial VMAT single-arc plans were remarkable; here, only 21% of all plans were acceptable regarding the PTV (in contrast to 68% for 2A). Similar to some HN cases, the V_95%_ values were statistically significant lower than in the corresponding IMRT plans, and the maximum dose was out of tolerance. The isodose lines indicated that in the target region, many small hot and cold spots were found (example in Figure 5). Again, the limiting factor may be the 1 cm leaf width. However, in contrast to the brain cases, a modulation with two arcs allowed for the effect to be compensated.

**Figure 6:**
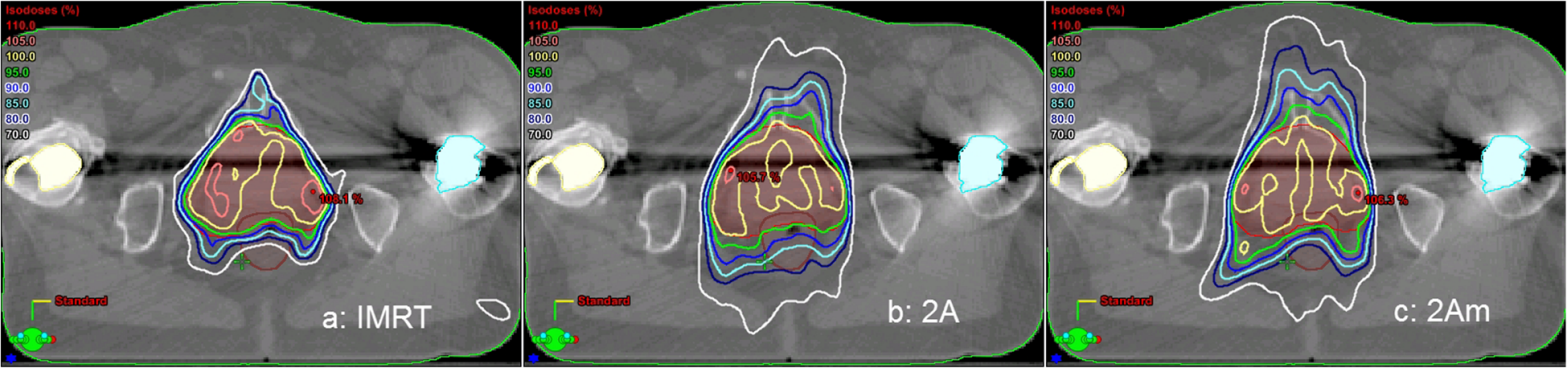
**Example of a prostate plan with two hip implants.** The implants are in yellow (left) and light blue (right), the rectum in brown, PTV in red; **(a)** IMRT: CN 0.81, rectum with V_40 Gy_ 38.9%, V_60 Gy_ 19.4%; bladder with V_40 Gy_ 31.1%, V_60 Gy_ 18.0%; **(b)** 2A: CN 0.79, rectum with V_40 Gy_ 62.4%, V_60 Gy_ 46.3%, bladder with V_40 Gy_ 38.4%, V_60 Gy_ 19.9%; **(c)** 2Am: CN 0.74, rectum with V_40 Gy_ 60.6%, V_60 Gy_ 33.8%, bladder with V_40 Gy_ 39.7%, V_60 Gy_ 21.1%.

Prostate plans including two hip implants must be considered separately: the clinical parameters (D_max_ and V_95%_ in the target, OAR sparing) for double-arc plans were acceptable, mostly after modification of the optimizing process. Before the modification, the sparing of the OAR (especially V_40 Gy_ and V_60 Gy_ of the rectum and V_60 Gy_ and V_70 Gy_ of the bladder) was out of tolerance and could be improved to passing rates of 81% for 2A plans and 63% for 1A plans. However, the conformity – for which no tolerance limit was given – lay statistically significant below the value of the IMRT plans before and after the modification (CN: 0.81 (IMRT), CN: 0.74 (VMAT), p: 0.03). The isodose lines of these plans clearly indicated that the dose-volume objectives were met with the values given in Table 1, but healthy tissue around the target was irradiated at a non-acceptable level (example in Figure 6).

As summarized in Tables 2 and 3 and shown in the detailed results for each tumor region, plans with two arcs provided a better plan quality than plans with one arc. The differences varied but were mainly significant and clinically important for the target coverage, particularly for the following: breast (D_max_: 114.1% [1A] vs. 109.4% [2A], V_95%_: 85.6% [1A] vs. 89.9% [2A]), prostate with one hip implant (V_95%_: 94.8% [1A] vs. 96.4% [2A]) and cervical plans (D_max_: 111.3% [1A] vs. 109.4% [2A], V_95%_: 91.9% vs. 95.2% [2A]). Clinically acceptable target dose coverage was difficult to achieve with a single-arc technique; however, it was possible with two arcs. Similarly, the OAR sparing was better for double-arc plans. However, the differences were often small and detectable even when statistically significant with no clinical relevance, e.g., D_max_ of the bladder for cervical cases: 47.8 Gy (1A) vs. 47.3 Gy (2A); D_max_ of the brainstem of 64 Gy-HN-cases: 41.8 Gy (1A) vs. 39.4 Gy (2A) and V_20 Gy_ of the lungs for breast cases: 10.0% (1A) vs. 9.5% (2A).

The integral body dose and the number of MUs were significantly different in favor of VMAT; the number of MUs was approximately 30% smaller compared to IMRT (except for prostate with hip implants, where the number of MUs was approximately the same). This value is comparable to previous studies on step-and-shoot IMRT that reported reductions of 12% to 20% [10,31] and studies on sliding-window IMRT that reported reductions of 40% to 55% [11,16]. As the number of MUs per treatment is correlated with the amount of scatter dose and leakage radiation, which could be important based on the induction of secondary malignancies [32,33], a decrease in the number of MUs achieved with VMAT is desirable. Furthermore, the evaluated plans had highly significant lower total integral doses (p < 0.001), which was represented by the mean dose to the patient body (VMAT: 15.3 Gy; IMRT 16.0 Gy). But the differences depend strongly on the tumor region: the smallest differences of 0.1 Gy were identified for HN cases with 64 Gy, and the largest differences (0.9 Gy) were identified for endometrial cases. These values are not consistent with the findings of some previous studies [10,20,34,35] that reported that the integral dose delivered to the patient is independent of the number of beams and the treatment technique; however, some other studies confirm our results [11,16,18]. In contrast, the total body volume receiving at least 5 Gy (V_5Gy_) was slightly larger for VMAT (+0.7% with p < 0.001) than for IMRT. This finding indicates that the integral dose for VMAT was smaller and mainly visible in the high-dose region; however, the low-dose region was slightly larger than for IMRT, possibly because the dose is spread all over the body, in contrast to IMRT, in which high doses are achieved only in the beam directions and hardly any doses are achieved outside of the beam directions.

## Conclusions

The results of this study have demonstrated that the quality achieved using the Varian Eclipse treatment planning system with single- and double-arc VMAT plans for Elekta linear accelerators is comparable to that of fixed-field step-and-shoot IMRT for a variety of tumor sites. Furthermore, plans with two arcs provide better dose distributions than plans with one arc. In addition, the template-based planning approach demonstrates that with minimal effort, clinically acceptable plans can be generated. Additionally, the reduced treatment time and smaller number of MUs are the major advantages of VMAT compared with IMRT. These results show that VMAT using Eclipse as TPS for Elekta linear accelerators is a valuable alternative to well-established IMRT technique in clinical routine.

## Competing interests

The authors declare that they have no competing interests.

## Authors’ contributions

SP is lead author, who designed the study and performed treatment planning, collected and analyzed data, interpreted data, revised literature and drafted the manuscript. HS and LP participated in the study design, in the discussion and interpretation of data and revised the manuscript critically. All authors read and approved the final manuscript.

## Supplementary Material

Additional file 1 List of the 10 used optimization templates. Click here for file

Additional file 2 Mean DVH and table with mean values for IMRT and VMAT comparison of head and neck cases with a prescription of 64 Gy. Click here for file

Additional file 3 Mean DVH and table with mean values for IMRT and VMAT comparison of breast cases. Click here for file

Additional file 4 Mean DVH and table with mean values for IMRT and VMAT comparison of prostate cases with lymph nodes and a prescription of 45 Gy. Click here for file

Additional file 5 Mean DVH and table with mean values for IMRT and VMAT comparison of prostate cases with one hip implant. Click here for file
