# Supplementary material for: A treatment planning study comparing Elekta VMAT and fixed field IMRT using the varian treatment planning system eclipse
Source: Radiat Oncol. 2014 Jul 10;9:153. doi: 10.1186/1748-717X-9-153 (PMC4107584; doi:10.1186/1748-717X-9-153)
Supplement: Additional file 1 — List of the 10 used optimization templates. [file 1748-717X-9-153-S1.pdf]

List of all optimizing templates

Cervical 45 Gy

Structure	*Type*	*Objective*	*Dose*	*Volume*	*Weight*
Avoid	Avoidance	Upper	4	50	500
Avoid	Avoidance	Upper	20	18	750
Avoid	Avoidance	Upper	38	3	750
Avoid	Avoidance	Upper	42	0	750
PTV Help	Avoidance	Upper	44	0	300
Bladder-PTV	Organ	Upper	25	50	500
Bladder-PTV	Organ	Upper	33	20	500
Bladder-PTV	Organ	Upper	38	5	500
Bladder-PTV	Organ	Upper	40	0	600
Rectum-PTV	Organ	Upper	33	20	500
Rectum-PTV	Organ	Upper	38	5	500
Rectum-PTV	Organ	Upper	40	0	600
Small intestine-PTV	Organ	Upper	21	52	300
Small intestine-PTV	Organ	Upper	33	20	300
Small intestine-PTV	Organ	Upper	40	0	300
PTV	PTV	Lower	44	100	950
PTV	PTV	Upper	46	0	999
PTV	PTV	mean	45	-	750

Head and Neck 64/54 Gy

Structure	*Type*	*Objective*	*Dose*	*Volume*	*Weight*
Avoid	Avoidance	Upper	18	40	800
Avoid	Avoidance	Upper	38	5	700
Avoid	Avoidance	Upper	53	0	700
Brain stem+3mm	Avoidance	Upper	50	0	700
Brain stem+5mm	Avoidance	Upper	52	0	800
PTV Help	Avoidance	Upper	53	0	300
Spine+3mm	Avoidance	Upper	37	0	850
Spine+5mm	Avoidance	Upper	38	0	900
Brain	Organ	Upper	50	0	600
Brain stem	Organ	Upper	48	0	450
Lips	Organ	Upper	40	0	800
Parotid lft	Organ	Upper	26	56	150
Parotid lft	Organ	Upper	45	36	150
Parotis rgt	Organ	Upper	27	55	150
Parotis rgt	Organ	Upper	46	35	150
Spine	Organ	Upper	36	0	600
PTV 54Gy	PTV	Lower	53	100	950
PTV 54Gy	PTV	Upper	55	0	800
PTV 54Gy	PTV	mean	54	-	900
PTV 64Gy	PTV	Lower	63	100	900
PTV 64Gy	PTV	Upper	65	0	999
PTV 64Gy	PTV	mean	64	-	850

Head and Neck 64/56 Gy

Structure	*Type*	*Objective*	*Dose*	*Volume*	*Weight*
Avoid	Avoidance	Upper	18	40	800
Avoid	Avoidance	Upper	38	5	700
Avoid	Avoidance	Upper	55	0	700
Brain stem+3mm	Avoidance	Upper	50	0	700
Brain stem+5mm	Avoidance	Upper	52	0	800
PTV Help	Avoidance	Upper	55	0	300
Spine+3mm	Avoidance	Upper	37	0	850
Spine+5mm	Avoidance	Upper	38	0	900
Brain	Organ	Upper	50	0	600
Brain stem	Organ	Upper	48	0	450
Lips	Organ	Upper	40	0	800
Parotid lft	Organ	Upper	26	56	150
Parotid lft	Organ	Upper	45	36	150
Parotis rgt	Organ	Upper	27	55	150
Parotis rgt	Organ	Upper	46	35	150
Spine	Organ	Upper	36	0	600
PTV 56Gy	PTV	Lower	55	100	950
PTV 56Gy	PTV	Upper	57	0	800
PTV 56Gy	PTV	mean	56	-	900
PTV 64Gy	PTV	Lower	63	100	900
PTV 64Gy	PTV	Upper	65	0	999
PTV 64Gy	PTV	mean	64	-	850

Head and Neck 69.3/54 Gy

Structure	*Type*	*Objective*	*Dose*	*Volume*	*Weight*
Avoid	Avoidance	Upper	20	40	800
Avoid	Avoidance	Upper	38	5	700
Avoid	Avoidance	Upper	53	0	700
Brain stem+3mm	Avoidance	Upper	52	0	800
Brain stem+5mm	Avoidance	Upper	50	0	700
PTV Help	Avoidance	Upper	53	0	300
Spine+3mm	Avoidance	Upper	37	0	850
Spine+5mm	Avoidance	Upper	38	0	900
Brain	Organ	Upper	50	0	600
Brain stem	Organ	Upper	48	0	450
Lips	Organ	Upper	40	0	800
Parotid lft	Organ	Upper	26	56	150
Parotid lft	Organ	Upper	45	36	150
Parotis rgt	Organ	Upper	27	55	150
Parotis rgt	Organ	Upper	46	35	150
Spine	Organ	Upper	36	0	600
PTV 54Gy	PTV	Lower	53	100	950
PTV 54Gy	PTV	Upper	55	0	800
PTV 54Gy	PTV	mean	54	-	900

PTV 69Gy	PTV	Lower	68.3	100	900
PTV 69Gy	PTV	Upper	70.3	0	999
PTV 69Gy	PTV	mean	69.3	-	850

Head and Neck 69.3/56.1 Gy

Structure	*Type*	*Objective*	*Dose*	*Volume*	*Weight*
Avoid	Avoidance	Upper	22	22	700
Avoid	Avoidance	Upper	38	5	700
Avoid	Avoidance	Upper	55.1	0	700
Brain stem+3mm	Avoidance	Upper	50	0	700
Brain stem+5mm	Avoidance	Upper	52	0	800
PTV Help	Avoidance	Upper	55.1	0	300
Spine+3mm	Avoidance	Upper	32	0	925
Spine+5mm	Avoidance	Upper	34	0	900
Brain	Organ	Upper	50	0	600
Brain stem	Organ	Upper	48	0	450
Lips	Organ	Upper	40	0	800
Parotid lft	Organ	Upper	26	56	150
Parotid lft	Organ	Upper	45	36	150
Parotis rgt	Organ	Upper	27	55	150
Parotis rgt	Organ	Upper	46	35	150
Spine	Organ	Upper	31	0	950
PTV 56Gy	PTV	Lower	55.1	100	900
PTV 56Gy	PTV	Upper	57.1	0	850
PTV 56Gy	PTV	mean	56.1	-	950
PTV 69Gy	PTV	Lower	68.3	100	900
PTV 69Gy	PTV	Upper	70.3	0	999
PTV 69Gy	PTV	mean	69.3	-	900

Head and Neck 69.3/56.1/52.8 Gy

Structure	*Type*	*Objective*	*Dose*	*Volume*	*Weight*
Avoid	Avoidance	Upper	22	22	700
Avoid	Avoidance	Upper	43	8	700
Avoid	Avoidance	Upper	51.8	0	700
Brain stem+3mm	Avoidance	Upper	52	0	800
Brain stem+5mm	Avoidance	Upper	50	0	700
PTV Help	Avoidance	Upper	51.8	0	300
Spine+3mm	Avoidance	Upper	32	0	925
Spine+5mm	Avoidance	Upper	34	0	900
Brain	Organ	Upper	50	0	600
Brain stem	Organ	Upper	48	0	450
Lips	Organ	Upper	40	0	800
Parotid lft	Organ	Upper	26	56	200
Parotid lft	Organ	Upper	45	36	200
Parotis rgt	Organ	Upper	27	55	200
Parotis rgt	Organ	Upper	46	35	200
Spine	Organ	Upper	31	0	950
PTV 52.8Gy	PTV	Lower	51.8	100	950
PTV 52.8Gy	PTV	Upper	53.8	0	900
PTV 52.8Gy	PTV	mean	52.8	-	950

PTV 56.1Gy	PTV	Lower	55.1	100	990
PTV 56.1Gy	PTV	Upper	57.1	0	900
PTV 56.1Gy	PTV	mean	56.1	-	850
PTV 69.3Gy	PTV	Lower	68.3	100	900
PTV 69.3Gy	PTV	Upper	70.3	0	999
PTV 69.3Gy	PTV	mean	69.3	-	850

Breast 50 Gy

Structure	*Type*	*Objective*	*Dose*	*Volume*	*Weight*
Avoid	Avoidance	Upper	14	19	600
Avoid	Avoidance	Upper	30	4	600
Avoid	Avoidance	Upper	47	0	700
PTV Help	Avoidance	Upper	49	0	300
Heart	Organ	Upper	21	16	700
Lung both	Organ	Upper	14	9	500
Lung contr	Organ	Upper	10	20	950
Lung ips	Organ	Upper	10	24	800
Lung ips	Organ	Upper	15	19	990
Spine	Organ	Upper	22	0	500
PTV Mamma li	PTV	Lower	49	100	800
PTV Mamma li	PTV	Upper	51	0	999
PTV Mamma li	PTV	mean	50	-	850

Prostate LN 45 Gy

Structure	*Type*	*Objective*	*Dose*	*Volume*	*Weight*
Avoid	Avoidance	Upper	10	56	500
Avoid	Avoidance	Upper	21	21	750
Avoid	Avoidance	Upper	38	3	750
Avoid	Avoidance	Upper	42	0	850
PTV Help	Avoidance	Upper	44	0	300
Bladder-PTV	Organ	Upper	22	55	600
Bladder-PTV	Organ	Upper	24	40	600
Bladder-PTV	Organ	Upper	33	20	600
Bladder-PTV	Organ	Upper	40	0	800
Femur head lft	Organ	Upper	30	0	300
Femur head rgt	Organ	Upper	31	0	300
Rectum-PTV	Organ	Upper	18	67	600
Rectum-PTV	Organ	Upper	24	41	600
Rectum-PTV	Organ	Upper	30	17	600
Rectum-PTV	Organ	Upper	40	0	850
Small intestine-PTV	Organ	Upper	21	52	300
Small intestine-PTV	Organ	Upper	33	20	300
Small intestine-PTV	Organ	Upper	40	0	300
PTV	PTV	Lower	44	100	950
PTV	PTV	Upper	46	0	999
PTV	PTV	mean	45	-	750

Prostate 74 Gy

Structure	*Type*	*Objective*	*Dose*	*Volume*	*Weight*
Avoid	Avoidance	Upper	10	26	700
Avoid	Avoidance	Upper	36	7	700
Avoid	Avoidance	Upper	70	0	800
PTV Help	Avoidance	Upper	73	0	300
Bladder-PTV	Organ	Upper	10	47	600
Bladder-PTV	Organ	Upper	28	32	600
Bladder-PTV	Organ	Upper	60	11	600
Bladder-PTV	Organ	Upper	70	0	900
Femur head lft	Organ	Upper	11	6	50
Femur head rgt	Organ	Upper	10	7	50
Rectum	Organ	Upper	39	59	950
Rectum-PTV	Organ	Upper	41	54	600
Rectum-PTV	Organ	Upper	62	16	600
Rectum-PTV	Organ	Upper	67	10	600
Rectum-PTV	Organ	Upper	70	0	900
PTV	PTV	Lower	73	100	950
PTV	PTV	Upper	75	0	950
PTV	PTV	mean	74	-	850

Brain 60Gy

Structure	*Type*	*Objective*	*Dose*	*Volume*	*Weight*
Avoid	Avoidance	Upper	18	40	800
Avoid	Avoidance	Upper	38	5	700
Avoid	Avoidance	Upper	59	0	700
Brain stem+3mm	Avoidance	Upper	52	0	800
Brain stem+5mm	Avoidance	Upper	50	0	700
PTV Help	Avoidance	Upper	59	0	300
Spine+3mm	Avoidance	Upper	37	0	850
Spine+5mm	Avoidance	Upper	38	0	900
Brain	Organ	Upper	50	0	600
Brain stem	Organ	Upper	48	0	850
Chiasm	Organ	Upper	45	0	880
Lens lft	Organ	Upper	4.5	0	990
Lens rgt	Organ	Upper	4.5	0	990
Lips	Organ	Upper	40	0	800
Optical. Nerve lft	Organ	Upper	39	0	850
Optical. Nerve rgt	Organ	Upper	39	0	850
Parotid lft	Organ	Upper	26	56	150
Parotid lft	Organ	Upper	45	36	150

Parotis rgt	Organ	Upper	27	55	150
Parotis rgt	Organ	Upper	46	35	150
Spine	Organ	Upper	36	0	600
PTV	PTV	Lower	59	100	900
PTV	PTV	Upper	61	0	999
PTV	PTV	mean	60	-	850

Avoid: a helping structure built by adding 5 cm in all dimesions around PTV, substracting PTV, PTV Help and all other structures
PTV Help: a helping structure built by adding 5 mm in all directions aroung the PTV, subtracting again the PTV. Plans with multiple PTVs, PTV Help encloses all PTVs
