# Supplementary material for: A treatment planning study comparing Elekta VMAT and fixed field IMRT using the varian treatment planning system eclipse
Source: Radiat Oncol. 2014 Jul 10;9:153. doi: 10.1186/1748-717X-9-153 (PMC4107584; doi:10.1186/1748-717X-9-153)
Supplement: Additional file 2 — Mean DVH and table with mean values for IMRT and VMAT comparison of head and neck cases with a prescription of 64 Gy. [file 1748-717X-9-153-S2.pdf]

Comparison between IMRT and VMAT for 18 head and neck plans with a prescription of 64 Gy. Single and double arc plans before (1A, 2A) and after modification (1Am, 2Am) of the optimization penalties; values are expressed as the mean (range).

	IMRT *(n=18)*	1A *(n=18)*	1Am *(n=18)*	2A *(n=18)*	2Am *(n=18)*
PTV 64Gy					
D_{max} [%]	108.8 (106.2 - 113.2)	107.3 (104.8-115.4)[a+b+]	107.0 (104.8-112.2)[a+c+]	105.9 (104.4 -109.0)[a+b+]	106.1 (104.3 - 108.7[)a+c+]
$V_{95\%}$ [%]	90.7 (83.6 - 98.1)	92.9 (82.6 - 98.8)[b+]	92.6 (85.7 - 97.5)[c+]	95.3 (91.3 - 99.3)[a+b+]	94.9 (89.2 - 99.4)[a+c+]
HI	1.13 (1.07 - 1.19)	1.10 (1.08 - 1.20)[ab+]	1.11 (1.08 - 1.17)[c+]	1.09 (1.07 - 1.12)[a+b+]	1.09 (1.07 - 1.12)[a+c+]
CN	0.72 (0.23 - 0.83)	0.79 (0.46 - 0.88)[ab+]	0.80 (0.46 - 0.88)a[+c+]	0.83 (0.47 - 0.91)[a+b+]	0.84 (0.47 - 0.90)[a+c+]
PTV54/56Gy					
D_{max} [%]	120.2 (111.2 - 127.8)	115.9 (111.6-125.3)[a+b+]	114.5 (111.1-122.1)[a+c+]	113.9 (108.8-121.8)[a+b+]	113.8 (108.3 - 121.8)[a+c+]
$V_{95\%}$ [%]	91.7 (84.1 - 96.9)	97.5 (93.7 - 99.5)[ab+]	94.4 (88.0 - 98.8)[ac+]	95.4 (89.3 - 98.4)[a+b+]	96.6 (90.0 - 99.3)[a+c+]
HI	1.15 (1.09 - 1.22)	1.12 (1.08 - 1.16)[ab+]	1.12 (1.08 - 1.16)[ac+]	1.10 (1.06 - 1.13)[a+b+]	1.10 (1.07 - 1.13)[a+c+]
CN	0.62 (0.44 - 0.74)	0.65 (0.38 - 0.75)[b+]	0.65 (0.40 - 0.75)[c+]	0.67 (0.44 - 0.76)[a+b+]	0.67 (0.44 - 0.77)[ac+]
$D_{50\%}$ [%]	100.4 (98.1 - 103.9)	101.2 (100.3 - 103.1)[b+]	100.8 (99.5 - 103.6)[c+]	100.6 (99.8 - 102.2)[b+]	100.3 (99.0 - 101.1)[ac+]
Body					
D_{mean} [Gy]	4.5 (2.3 - 7.2)	4.4 (2.3 - 6.5)[b+]	4.4 (2.3 - 6.7)	4.4 (2.3 - 6.5)[b+]	4.4 (2.3 - 6.5)[a]
V_{5Gy} [%]	14.9 (8.9 - 22.0)	15.2 (8.8 - 21.2)[b+]	15.1 (8.8 - 21.3)[c+]	15.5 (8.8 - 21.7)[a+b+]	15.4 (8.8 - 21.3)[ac+]
Brain					
D_{max} [Gy]	38.7 (4.0 - 62.1)	42.3 (5.8 - 54.2)[b+]	41.9 (4.8 - 56.3)[c+]	40.3 (6.4 - 53.6)[b+]	40.1 (5.6 - 55.7)[ac+]
Brain stem					
D_{max} [Gy]	35.3 (15.2 - 45.3)	42.7 (17.6 - 51.4)[a+]	41.8 (15.3 - 51.2)[a+c]	40.5 (14.7 - 52.2)[a]	39.4 (17.2 - 47.5)[c]
Lips					
D_{max} [Gy]	34.7 (23.1 - 59.6)	28.7 (15.3 - 54.4)[a+]	30.1 (15.3 - 55.3)[a]	28.8 (13.4 - 55.0)[a+]	30.7 (13.4 - 54.7)
Parotids					
D_{mean} [Gy]	30.1 (7.4 - 55.4)	30.9 (10.6 - 48.9)	30.4 (10.6 - 54.4)	30.6 (9.6 - 46.2	30.2 (10.3 - 53.0)
V_{28Gy} [%]	44.5 (0.0 - 100.0)	45.9 (0.0 - 89.4)[b]	43.4 (0.0 - 100.0)	44.6 (0.0 - 81.6)[b]	42.8 (0.0 - 98.9)[a]
Parotid lft					
D_{mean} [Gy]	29.5 (7.4 - 53.3)	29.8 (10.6 - 44.4)	29.4 (10.6 - 51.8)	29.7 (11.4 - 44.3)	29.3 (11.4 - 50.4)
V_{28Gy} [%]	45.4 (0.0 - 100.0)	44.9 (0.0 - 89.4)	42.8 (0.0 - 100.0)	43.7 (0.0 - 79.5)	42.3 (0.0 - 98.9)
Parotid rgt					
D_{mean} [Gy]	30.7 (8.1 - 55.4)	31.9 (11.2 - 48.9)	31.3 (10.6 - 54.4)	31.4 (9.6 - 46.2)	31.1 (10.3 - 53.0)
V_{28Gy} [%]	43.6 (0.0 - 100.0)	46.9 (0.0 - 87.1)	44.1 (0.0 - 99.4)	45.5 (0.0 - 81.6)	43.2 (0.0 - 98.7)
Spine					
D_{max} [Gy]	36.2 (30.6-44.9)	37.5 (33.4 - 45.3)[b+]	36.8 (32.1 - 44.1)	36.5 (32.4 - 42.5)[b+]	36.3 (32.9 - 41.3)[a]
MU	519.3 (307 - 847)	326.1 (295 - 406)[a+b+]	335.7 (285 - 388)[a+]	343.6 (303 - 444)[a+b+]	347.4 (282 - 472)[a+]

[a]p<0.05 for Wilcoxon matched-pair signed rank test vs. IMRT; [b]p<0.05 1A vs. 2A; [c]p<0.05 1Am vs. 2Am; [+]p<0.01.

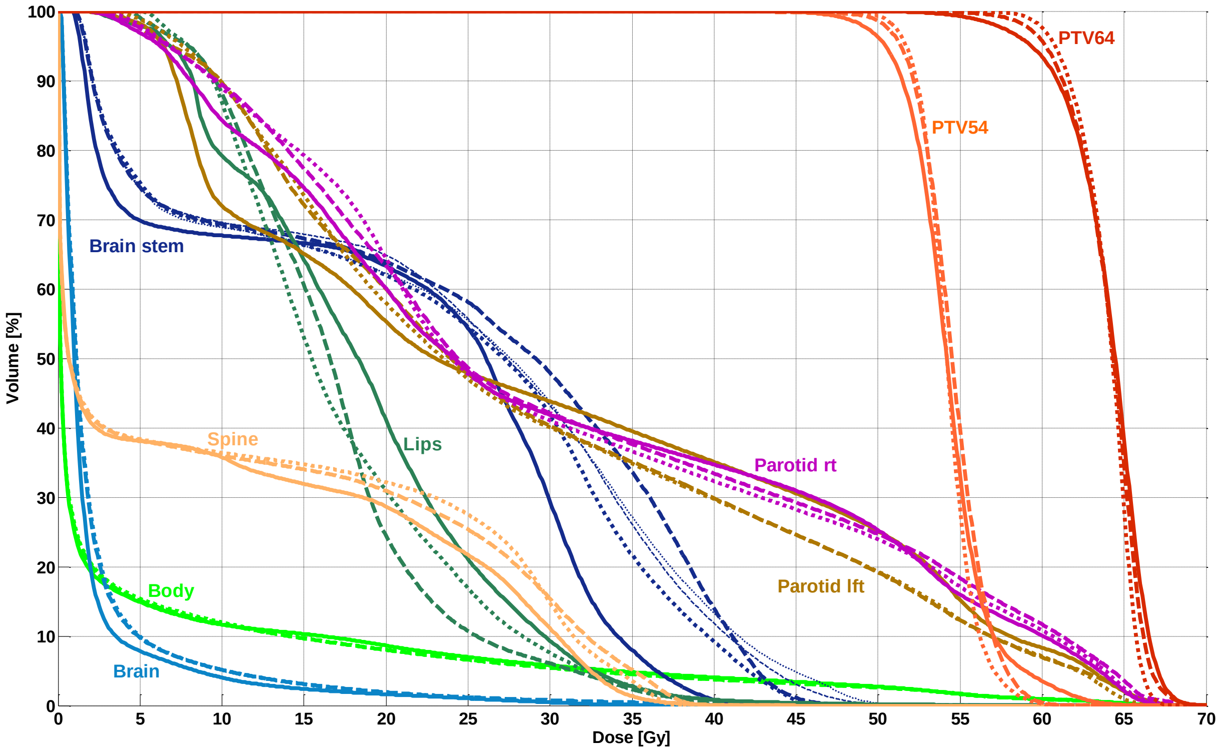

Mean DVH of 18 HN cancer cases with 64 Gy. Solid line: IMRT; thin dashed line: 1A; thin dotted line: 2A; fat dashed line: 1Am; fat dotted line: 2Am.
