# Supplementary material for: A treatment planning study comparing Elekta VMAT and fixed field IMRT using the varian treatment planning system eclipse
Source: Radiat Oncol. 2014 Jul 10;9:153. doi: 10.1186/1748-717X-9-153 (PMC4107584; doi:10.1186/1748-717X-9-153)
Supplement: Additional file 3 — Mean DVH and table with mean values for IMRT and VMAT comparison of breast cases. [file 1748-717X-9-153-S3.pdf]

Comparison between IMRT and VMAT for 10 breast cases. Single and double arc plans before (1A, 2A) and after modification (1Am, 2Am) of the optimization penalties; values are expressed as the mean (range).

	IMRT *(n=10)*	1A *(n=10)*	1Am *(n=10)*	2A *(n=10)*	2Am *(n=10)*
PTV					
D_{max} [%]	116.7 (112.7 - 120.3)	113.4 (110.4 - 119.9)[ab+]	114.1 (111.0 - 118.2)[c+]	109.2 (107.1 - 111.3)[a+b+]	109.4 (107.1 - 111.3)[a+c+]
$V_{95\%}$ [%]	86.8 (83.3 - 90.5)	85.6 (81.1 - 88.6)	85.8 (81.8 - 88.7)	89.5 (86.2 - 94.2)ab	89.8 (87.3 - 94.2)[a+]
HI	1.17 (1.13 - 1.20)	1.18 (1.15 - 1.24)[b+]	1.17 (1.15 - 1.22)[c+]	1.14 (1.09 - 1.17)[a+b+]	1.13 (1.09 - 1.17)[a+c+]
CN	0.74 (0.59 - 0.82)	0.77 (0.69 - 0.81)[b+]	0.74 (0.60 - 0.82)[c+]	0.80 (0.62 - 0.86)[b+]	0.80 (0.62 - 0.86)[c+]
Body					
D_{mean} [Gy]	9.5 (7.4 - 11.1)	9.0 (8.1 - 10.2)[b+]	9.1 (8.0 - 10.6)	9.3 (8.2 - 10.4)[b+]	9.3 (8.2 - 10.4)
V_{5Gy} [%]	44.6 (29.8 - 55.6)	40.5 (32.4 - 49.1)[b+]	41.7 (32.4 - 51.4)	43.3 (35.5 - 51.3)[b+]	43.3 (35.5 - 51.3)
Heart					
D_{mean} [Gy]	15.9 (9.7 - 23.4)	12.6 (5.7 - 17.8)[a]	12.8 (5.7 - 18.5)[a]	13.6 (8.8 - 18.1)	13.5 (8.8 - 17.9)
$D_{mean, left}$[Gy]	18.5 (15.1 – 23.4)	12.7 (10.8 – 15.3)	12.8 (10.8 – 15.3)	14.0 (12.0 – 17.3)	14.0 (12.0 – 17.3)
$D_{mean, rgt}$ [Gy]	12.6 (5.7 – 16.8)	12.4 (5.7 - 17.8)	12.7 (5.7 - 18.5)	13.0 (8.8 - 18.1)	12.9 (8.8 - 17.9)
Lung ips.					
V_{5Gy} [%]	88.7 (65.4 - 99.8)	81.2 (69.0 - 93.5)[a]	84.2 (73.2 - 95.1)	81.2 (70.3 - 89.2)[a]	81.5 (70.3 - 89.7)[a]
V_{20Gy} [%]	20.7 (18.7 - 25.4)	19.2 (15.8 - 21.6)[b+]	18.9 (14.1 - 21.6)	18.3 (16.0 - 20.4)[a+b+]	18.2 (16.0 - 20.4)[a+]
D_{mean} [Gy]	14.6 (12.2 - 17.6)	13.0 (11.0 - 14.7)[a]	13.0 (10.4 - 14.7)	12.6 (11.8 - 13.5)[a]	12.7 (11.8 - 13.5)[a]
Lung contr.					
V_{5Gy} [%]	54.0 (13.9 - 84.8)	47.3 (9.1 - 67.6)	50.0 (9.1 - 69.6)	56.1 (24.5 - 77.3)	55.4 (24.5 - 77.3)
V_{20Gy} [%]	1.5 (0.0 - 3.8)	1.4 (0.0 - 5.1)	1.9 (0.0 - 5.1)	1.5 (0.0 - 4.3)	1.6 (0.0 - 5.2)
D_{mean} [Gy]	6.3 (3.2 - 9.8)	5.9 (2.9 - 8.6)	6.2 (2.9 - 8.7)	6.5 (4.1 - 8.5)	6.5 (4.1 - 8.6)
Lungs					
V_{5Gy} [%]	71.4 (37.7 - 91.1)	63.6 (46.7 - 74.4)	66.4 (46.7 - 80.4)	68.4 (54.0 - 81.0)	68.2 (54.0 - 81.0)
V_{20Gy} [%]	10.6 (8.5 - 12.2)	9.9 (7.8 - 11.8)[b+]	10.0 (7.8 - 11.5)[c+]	9.4 (7.4 - 11.5)[ab+]	9.5 (7.4 - 11.8)[ac+]
D_{mean} [Gy]	10.3 (7.4 - 12.4)	9.3 (8.3 - 10.5)[a]	9.5 (8.3 - 10.7)	9.4 (8.3 - 10.6)	9.5 (8.3 - 10.8)
Spine					
D_{max} [Gy]	16.1 (7.9 - 22.4)	18.0 (7.1 - 25.6)	18.0 (7.1 - 25.9)	17.2 (11.5 - 24.5)	17.2 (11.5 - 24.9)
MU	699.3 (556 - 1123)	455.7 (380 - 529)[a+b]	507.2 (428 - 633)[a+]	472.3 (393 - 524)[a+b]	484.8 (393 - 609)[a+]

[a]p<0.05 for Wilcoxon matched-pair signed rank test vs. IMRT; [b]p<0.05 1A vs. 2A; [c]p<0.05 1Am vs. 2Am; [+]p<0.01.

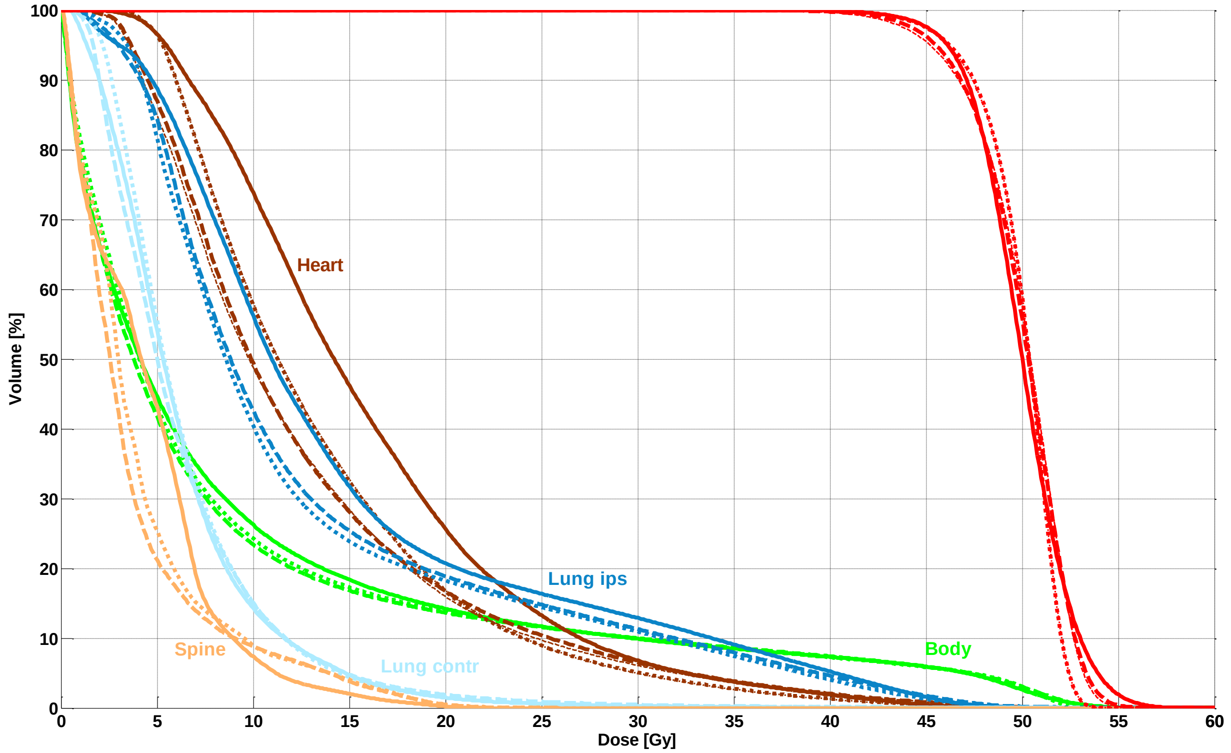

Mean DVH of 10 breast cancer cases. Solid line: IMRT; thin dashed line: 1A; thin dotted line: 2A; fat dashed line: 1Am; fat dotted line: 2Am.
