# Supplementary material for: A treatment planning study comparing Elekta VMAT and fixed field IMRT using the varian treatment planning system eclipse
Source: Radiat Oncol. 2014 Jul 10;9:153. doi: 10.1186/1748-717X-9-153 (PMC4107584; doi:10.1186/1748-717X-9-153)
Supplement: Additional file 4 — Mean DVH and table with mean values for IMRT and VMAT comparison of prostate cases with lymph nodes and a prescription of 45 Gy. [file 1748-717X-9-153-S4.pdf]

Comparison between IMRT and VMAT for 11 prostate cases including lymph nodes and a prescription of 45 Gy. Single and double arc plans before (1A, 2A) and after modification (1Am, 2Am) of the optimization penalties; values are expressed as the mean (range).

	IMRT *(n=11)*	1A *(n=11)*	1Am *(n=11)*	2A *(n=11)*	2Am *(n=11)*
PTV					
D_{max} [%]	110.2 (106.5 - 112.4)	108.3 (106.8 -12.5)[a+b+]	108.1 (106.8 -10.3)[a+c+]	106.7 (105.7 -07.7)a[+b+]	106.7 (105.7-107.7)[a+c+]
$V_{95\%}$ [%]	96.6 (93.3 - 98.6)	95.8 (92.9 - 98.9)[b+]	96.1 (92.2 - 98.9)[c+]	98.5 (96.1 - 99.9)[a+b+]	98.5 (96.1 - 99.9)[a+c+]
HI	1.09 (1.07 - 1.11)	1.09 (1.07 - 1.11)[b+]	1.09 (1.07 - 1.11)[c+]	1.06 (1.05 - 1.08)[a+b+]	1.06 (1.05 - 1.08)[a+c+]
CN	0.73 (0.62 - 0.83)	0.79 (0.73 - 0.86)[a+b+]	0.79 (0.73 - 0.86)[a+c+]	0.83 (0.76 - 0.87)[a+b+]	0.83 (0.76 - 0.87)[a+c+]
Body					
D_{mean} [Gy]	11.1 (7.9 - 15.2)	10.6 (7.5 - 14.5)[a+]	10.6 (7.5 - 14.3)[a+]	10.5 (7.4 - 14.4)[a+]	10.5 (7.4 - 14.4)[a+]
V_{5Gy} [%]	48.2 (33.7 - 67.5)	50.8 (35.4 - 69.4)[a+b]	50.6 (35.4 - 69.3)[a+c+]	51.0 (35.4 - 69.3)[a+b]	51.0 (35.4 - 69.3)[a+c+]
Rectum					
V_{40Gy} [%]	24.1 (10.7 - 42.1)	22.7 (11.0 - 44.6)	22.6 (11.0 - 44.6)	22.9 (11.4 - 45.3)	22.9 (11.4 - 45.3)
D_{max} [Gy]	47.8 (45.1 - 49.0)	46.8 (45.8 - 47.8)[a+]	47.0 (45.8 - 48.2)[a]	47.0 (46.3 - 47.7)[a]	47.0 (46.3 - 47.7)[a]
Bladder					
V_{40Gy} [%]	27.9 (11.9 - 50.4)	28.7 (7.2 - 50.0)	28.7 (7.2 - 50.0)	28.9 (7.2 - 50.6)	28.9 (7.2 - 50.6)
D_{max} [Gy]	48.2 (44.8 - 49.8)	47.8 (46.8 - 49.4)[b]	47.8 (46.8 - 48.9)[ac]	47.4 (46.6 - 48.2)[b]	47.4 (46.6 - 48.2)[c]
Small Intestine					
V_{45Gy} [ccm	1.6 (0.0 - 9.8)	1.2 (0.0 - 5.6)	2.1 (0.0 - 13.1)	1.2 (0.0 - 4.9)	1.2 (0.0 - 4.9)
D_{mean} [Gy]	17.3 (1.7 - 27.2)	16.2 (1.9 - 26.6)[a]	16.4 (1.9 - 24.6)	17.6 (1.8 - 26.8)	17.6 (1.8 - 26.8)
Femur heads					
D_{max} [Gy]	38.7 (30.1 - 45.8)	35.6 (27.9 - 38.2)[ab]	35.3 (27.9 - 38.2)[a+]	33.7 (29.5 - 38.7)[a+b]	33.7 (29.5 - 38.7)[a+]
D_{mean} [Gy]	23.8 (17.7 - 27.0)	23.5 (18.5 - 29.0)	23.1 (18.5 - 29.0)	21.9 (18.6 - 26.1)	21.9 (18.6 - 26.1)
MU	744.3 (591 - 925)	463.1 (392 - 577)[a+b+]	480.8 (392 - 595)[a+c+]	543.5 (474 - 609)[a+b+]	543.5 (474 - 609)[a+c+]

[a]p<0.01 for Wilcoxon matched-pair signed rank test vs. IMRT; [b]p<0.01 1A vs. 2A; [c]p<0.01 1Am vs. 2Am.

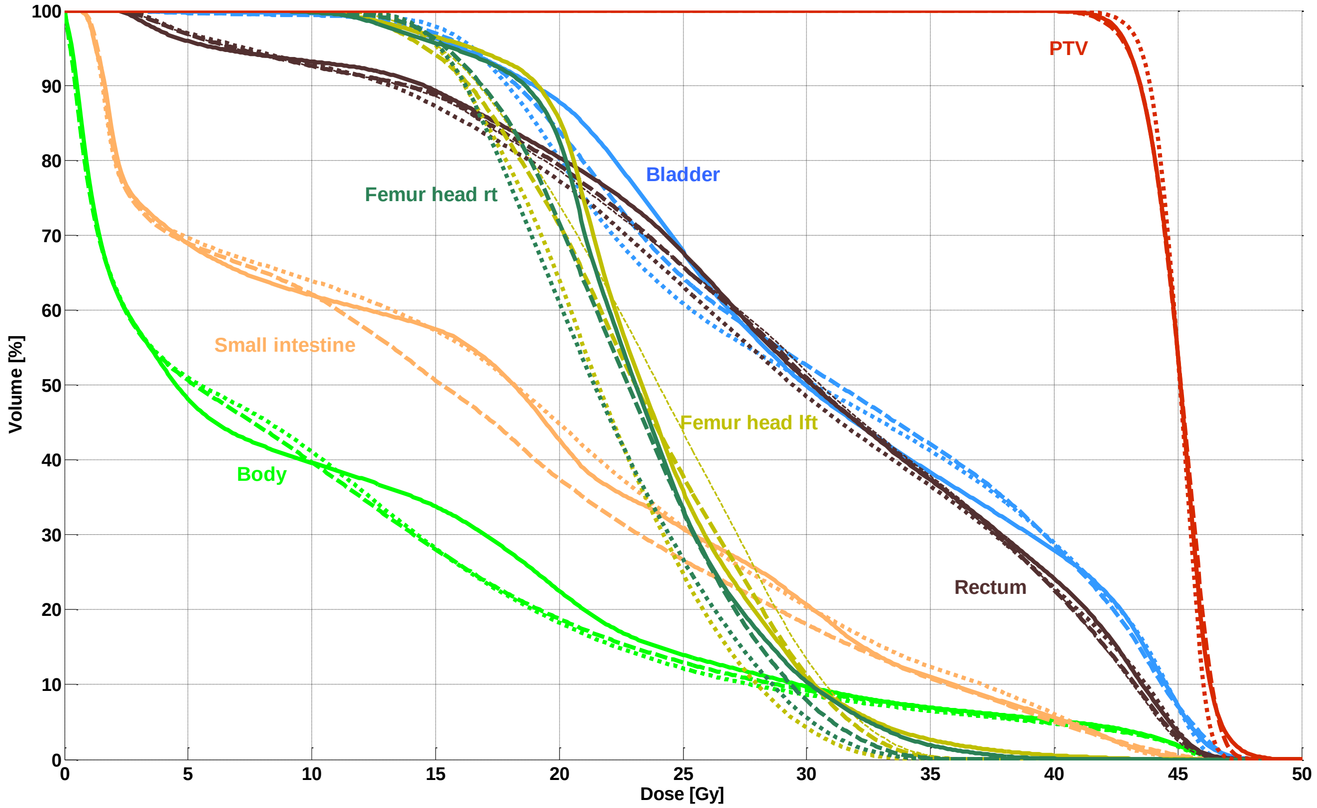

Mean DVH of 16 prostate cancer cases with lymph nodes. Solid line: IMRT; thin dashed line: 1A; thin dotted line: 2A; fat dashed line: 1Am; fat dotted line: 2Am
