# Supplementary material for: A treatment planning study comparing Elekta VMAT and fixed field IMRT using the varian treatment planning system eclipse
Source: Radiat Oncol. 2014 Jul 10;9:153. doi: 10.1186/1748-717X-9-153 (PMC4107584; doi:10.1186/1748-717X-9-153)
Supplement: Additional file 5 — Mean DVH and table with mean values for IMRT and VMAT comparison of prostate cases with one hip implant. [file 1748-717X-9-153-S5.pdf]

Comparison between IMRT and VMAT for 16 prostate cases with one hip implant. Single and double arc plans before (1A, 2A) and after modification (1Am, 2Am) of the optimization penalties; values are expressed as the mean (range).

	IMRT *(n=16)*	1A *(n=16)*	1Am *(n=16)*	2A *(n=16)*	2Am *(n=16)*
PTV					
D_{max} [%]	106.4 (103.9 - 110.2)	108.2 (104.5 - 110.7)[ab+]	108.4 (104.9 - 111.3)[a+c+]	107.6 (104.3 - 110.9)[b+]	107.5 (104.2 - 110.1)[c+]
$V_{95\%}$ [%]	97.2 (93.0 - 99.8)	95.2 (89.5 - 99.7)[ab+]	94.8 (87.7 - 99.2)[ac+]	96.4 (90.2 - 99.9)[b+]	96.4 (90.0 - 99.6)[c+]
HI	1.08 (1.05 - 1.12)	1.09 (1.04 - 1.14)[a]	1.09 (1.05 - 1.14)[ac+]	1.08 (1.04 - 1.13)	1.08 (1.04 - 1.13)[c+]
CN	0.76 (0.67 - 0.90)	0.83 (0.77 - 0.89)[a+b]	0.83 (0.77 - 0.89)[a+c]	0.84 (0.73 - 0.90)[a+b]	0.84 (0.73 - 0.90)[a+c]
Body					
D_{mean} [Gy]	5.49 (2.85 - 9.48)	5.00 (2.79 - 7.57)[a+b]	5.0 (2.7 - 7.7)[a+c]	5.0 (2.7 - 7.8)[a+b]	5.0 (2.7 - 7.7)[a+c]
V_{5Gy} [%]	20.0 (10.5 - 35.5)	21.1 (11.95 - 30.37)[ab+]	21.1 (11.9 - 30.4)[ac+]	21.5 (12.1 - 31.9)[a+b+]	21.5 (12.0 - 32.1)[ac+]
Rectum					
V_{40Gy} [%]	49.9 (31.7 - 65.9)	56.0 (36.5 - 63.1)[a+]	54.7 (36.5 - 60.9)[ac]	56.5 (36.9 - 62.9)[a+]	56.1 (36.9 - 62.8)[a+c]
V_{60Gy} [%]	25.7 (15.7 - 43.0)	25.2 (11.2 - 38.7)	24.2 (11.2 - 33.8)	25.6 (14.1 - 34.8)	25.3 (14.1 - 32.9)
V_{70Gy} [%]	6.2 (0.0 - 15.2)	6.1 (0.0 - 12.9)	6.2 (0.0 - 12.7)	6.4 (0.0 - 13.8)	6.2 (0.0 - 13.1)
D_{max} [Gy]	73.2 (67.3 - 80.5)	74.7 (69.1 - 78.8)[a+]	75.0 (69.1 - 78.3)[a+c+]	74.4 (68.5 - 78.3)[a+]	74.3 (68.5 - 79.2)[a+c+]
Bladder					
V_{40Gy} [%]	35.2 (7.6 - 56.3)	32.4 (7.1 - 49.6)	32.6 (7.1 - 50.7)	32.4 (7.5 - 47.4)	32.1 (7.5 - 47.7)
V_{60Gy} [%]	18.3 (4.6 - 32.2)	15.5 (3.2 - 29.5)[a]	15.5 (3.2 - 29.2)[a+]	15.7 (3.4 - 27.8)[a+]	15.5 (3.4 - 27.8)[a]
V_{70Gy} [%]	5.0 (0.0 - 15.2)	4.7 (0.0 - 13.2)	4.6 (0.0 - 14.1)	4.9 (0.0 - 13.8)	4.7 (0.0 - 13.8)
D_{max} [Gy]	74.3 (67.2 - 81.2)	75.5 (69.7 - 80.0)[ab+]	75.3 (69.7 - 79.5)[c+]	75.0 (69.3 - 79.7)[b+]	74.8 (68.8 - 79.3)[c+]
Femur head					
D_{max} [Gy]	34.3 (24.1 - 45.0)	32.8 (20.1 - 46.6)	31.7 (19.0 - 53.9)	31.6 (17.2 - 47.4)	29.8 (15.4 - 48.4)[a]
D_{mean} [Gy]	11.6 (4.8 - 24.2)	15.2 (5.9 - 29.2)[a]	15.4 (4.2 - 35.3)[a]	15.2 (5.1 - 31.5)[a+]	14.7 (3.8 - 28.2)[a]
Implant					
D_{max} [Gy]	29.5 (14.0 - 73.4)	23.0 (11.1 - 41.4)[a]	23.0 (12.0 - 39.7)[a+]	22.8 (9.6 - 40.9)[a+]	23.1 (9.6 - 45.1)[a+]
D_{mean} [Gy]	5.7 (3.2 - 14.1)	4.9 (2.5 - 9.7)[a]	5.1 (2.5 - 10.1)[a]	5.1 (2.5 - 10.8)[a]	5.2 (2.5 - 11.4)
MU	559.6 (356 - 651)	570.9 (470 - 833)	568.6 (470 - 833)	587.3 (477 - 939)	574.1 (436 - 939)

[a]$p<0.05$ for Wilcoxon matched-pair signed rank test vs. IMRT; [b]$p<0.05$ 1A vs. 2A; [c]$p<0.05$ 1Am vs. 2Am; [+]$p<0.01$.

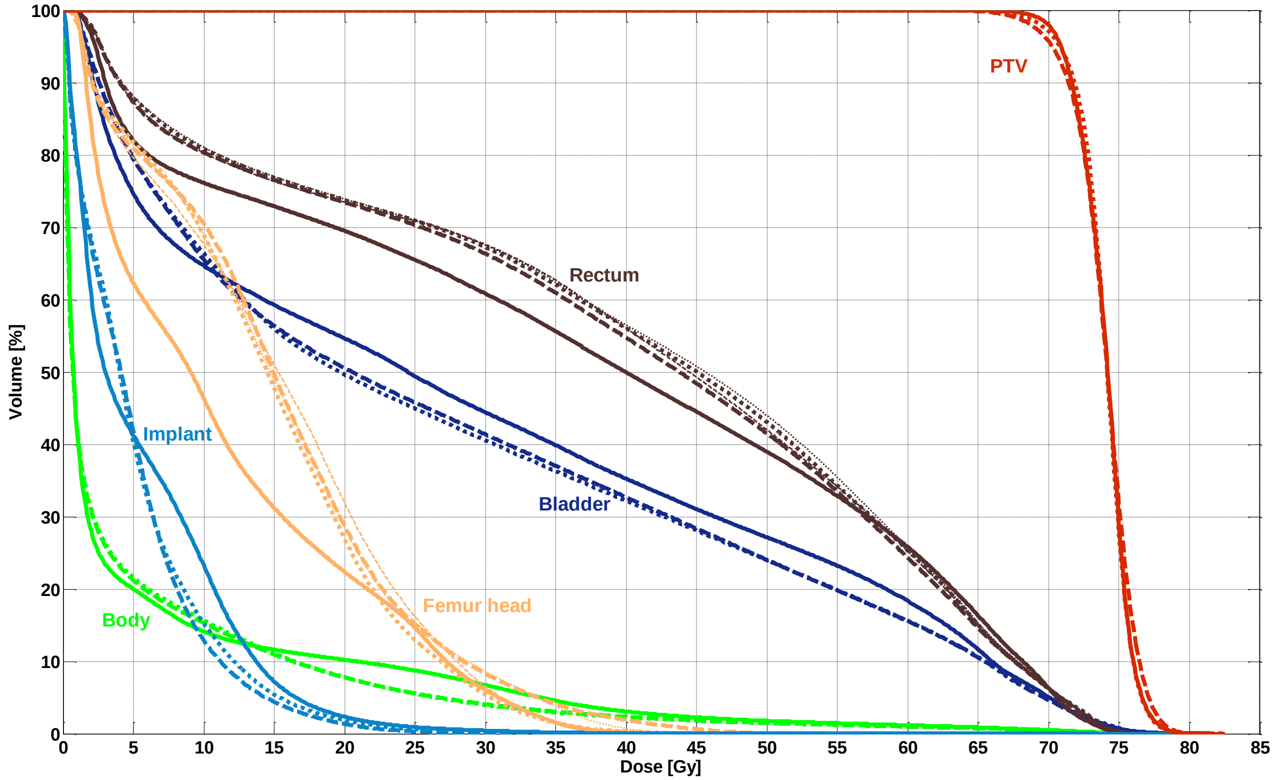

Mean DVH of 16 prostate cancer cases with one hip implants. Solid line: IMRT; thin dashed line: 1A; thin dotted line: 2A; fat dashed line: 1Am; fat dotted line: 2Am
